# Lateral Flow Immunoassays for SARS-CoV-2

**DOI:** 10.3390/diagnostics12112854

**Published:** 2022-11-18

**Authors:** Geik Yong Ang, Kok Gan Chan, Chan Yean Yean, Choo Yee Yu

**Affiliations:** 1 Faculty of Sports Science and Recreation, Universiti Teknologi MARA, Shah Alam 40450, Malaysia; 2 Division of Genetics and Molecular Biology, Institute of Biological Sciences, Faculty of Science, University of Malaya, Kuala Lumpur 50603, Malaysia; 3 International Genome Centre, Jiangsu University, Zhenjiang 212013, China; 4 Department of Medical Microbiology and Parasitology, School of Medical Sciences, Universiti Sains Malaysia, Kota Bharu 16150, Malaysia; 5 Laboratory of Vaccine and Biomolecules, Institute of Bioscience, Universiti Putra Malaysia, Serdang 43400, Malaysia

**Keywords:** dipstick, immunochromatography, antigen, antibody, diagnostic, serology, point-of-care, immunosensor

## Abstract

The continued circulation of SARS-CoV-2 virus in different parts of the world opens up the possibility for more virulent variants to evolve even as the coronavirus disease 2019 transitions from pandemic to endemic. Highly transmissible and virulent variants may seed new disruptive epidemic waves that can easily put the healthcare system under tremendous pressure. Despite various nucleic acid-based diagnostic tests that are now commercially available, the wide applications of these tests are largely hampered by specialized equipment requirements that may not be readily available, accessible and affordable in less developed countries or in low resource settings. Hence, the availability of lateral flow immunoassays (LFIs), which can serve as a diagnostic tool by detecting SARS-CoV-2 antigen or as a serological tool by measuring host immune response, is highly appealing. LFI is rapid, low cost, equipment-free, scalable for mass production and ideal for point-of-care settings. In this review, we first summarize the principle and assay format of these LFIs with emphasis on those that were granted emergency use authorization by the US Food and Drug Administration followed by discussion on the specimen type, marker selection and assay performance. We conclude with an overview of challenges and future perspective of LFI applications.

## 1. Introduction

It has been almost three years since the onset of the coronavirus disease 2019 (COVID-19) pandemic but global case incidence has remained high with over 2.9 million new cases and 8300 fatalities reported in the week ending on 2 October 2022 [1]. All these add to the staggering number of 615 million confirmed cases and 6.5 million deaths that were attributed to the novel severe acute respiratory syndrome coronavirus 2 (SARS-CoV-2) virus globally as of 2 October 2022 [1]. The virus can be transmitted from an infected person via infective respiratory droplets and fomites in the immediate environment [2]. Following a mean incubation period of 5 days, the most common clinical presentation of COVID-19 includes fever, cough, and shortness of breath with typical imaging features consisting of bilateral pneumonia, multiple mottling, and ground-glass opacity [3,4]. Although the majority of COVID-19 cases are mild, serious complications may develop in a subset of patients including acute respiratory distress syndrome, acute cardiac injury, acute kidney injury, and septic shock [4,5,6]. The disease can progress rapidly from mild to severe: the median times from onset of symptoms to intensive care unit admission and death were 10.5 days [5] and 14 days [7], respectively.

Given that COVID-19 affects all age groups with a spectrum of illnesses ranging from asymptomatic to fatal [6], the availability of rapid, sensitive and specific diagnostic tests that can accurately triage and identify COVID-19 patients at first point of contact will be central to concomitant measures to control the spread of this disease. The swift release of the SARS-CoV-2 genome sequence early in the outbreak had allowed highly specific and sensitive nucleic acid tests to be developed and used for diagnostic, screening and surveillance purposes. Presently, a range of nucleic acid amplification tests (NAATs) have been granted emergency use authorization (EUA) status by the US Food and Drug Administration (FDA). These include non-isothermal- and isothermal-based amplification technologies with various amplicon detection methods being employed such as fluorometric, colorimetric, electrochemical, magnetic resonance, clustered regularly interspaced short palindromic repeats/Cas systems, matrix-assisted laser desorption ionization time-of-flight and sequencing [8,9]. However, the technical intricacies and sophisticated instrument requirements of FDA-EUA nucleic acid tests confine most of them to Clinical Laboratory Improvement Amendments (CLIA)-certified, high-complexity laboratories.

Compared to nucleic acid tests, lateral flow immunoassays (LFIs) that detect SARS-CoV-2 antigen are more suited for decentralized testing to identify acute or early infection as they are relatively cheap to produce, easy to use, yield a rapid visual result and are virtually equipment-free. LFIs that fulfill the World Health Organization’s ASSURED criteria (Affordable, Sensitive, Specific, User-friendly, Rapid and robust, Equipment-free and Deliverable to end users) [10] can rapidly expand testing capabilities for this virus, particularly in middle- and low-income countries. At point-of-care settings, the on-site detection and same-day reporting features of these paper-based diagnostic tools will greatly help physicians in making evidence-based COVID-19 patient management decisions. On the other hand, serological LFIs that assess the immune status to COVID-19 by detecting antibodies against SARS-CoV-2 provides important epidemiological information such as the cumulative incidence of infection, the proportion of mild and asymptomatic cases, the proportion of severe and fatal cases among those who are infected, and the immune status of the population [11].

The merits of LFI make it an attractive first-line test against COVID-19 as this technology platform does not suffer from the many drawbacks associated with real-time reverse transcription polymerase chain reaction (RT-PCR) and enzyme-linked immunosorbent assay (ELISA), i.e., multiple liquid-handling steps, high equipment requirements and longer turnaround time. A general comparison between LFI, RT-PCR and ELISA is presented in Table 1. Although the applications of LFI for COVID-19 testing have been covered in recent reviews [12,13,14,15], there were no attempts to provide a comprehensive overview of LFIs that have received EUA from the US FDA. As of 11 October 2022, a total of 69 LFIs have been granted EUA: 44 are antigen diagnostic tests and the remaining 25 are serological tests that detect antibodies, such as immunoglobulin (Ig) M and/or IgG, against SARS-CoV-2. In this review, we discuss the principle and different assay formats of the LFIs that detect SARS-CoV-2 antigen(s) and anti-SARS-CoV-2 antibodies in detail. We also compare the performance of these LFIs and conclude with the challenges and future perspective of LFI applications beyond the COVID-19 pandemic.

## 2. LFI: An Overview

### 2.1. Principle of LFI

**Figure 1:**
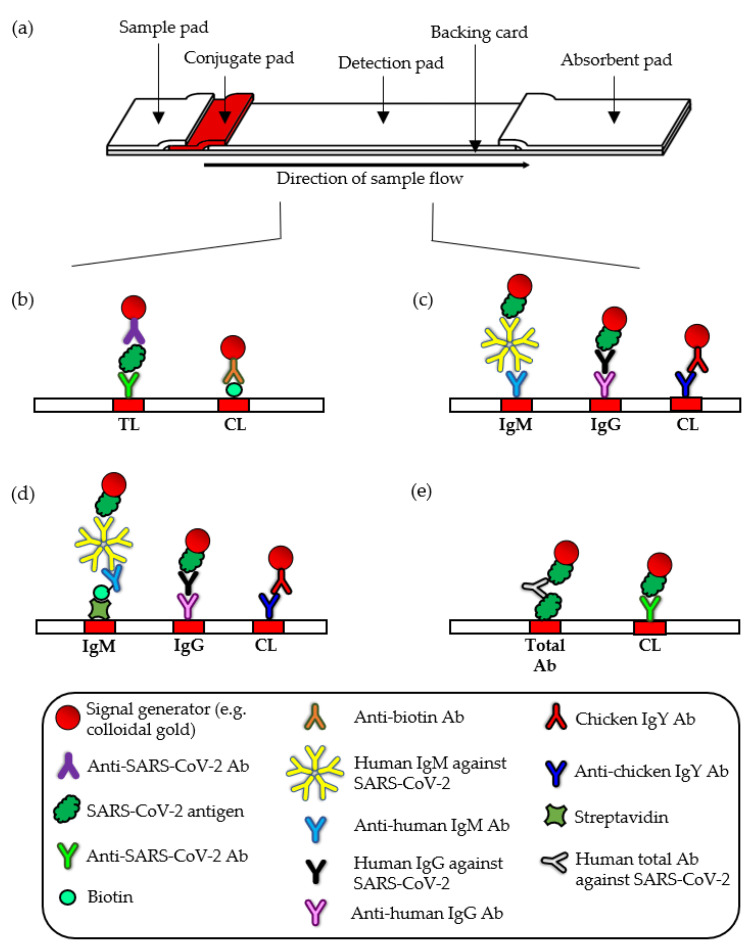
(**a**) Assembly of the different components of LFI. Assay formats for the detection of SARS-CoV-2 antigen (**b**), IgM and IgG (**c**,**d**), and total antibody (**e**). TL, test line; CL, control line; Ab, antibody.

LFIs are self-contained and self-operating devices: properties that are made possible by the LFI configuration and capillary action. By mounting several components of different materials (a sample pad, conjugate pad, detection pad and an absorbent pad) in an overlapping manner, a device that functions as a single test system is created wherein an aqueous medium can flow continuously from proximal to distal ends of the strip (Figure 1a). Briefly, when an aqueous biological sample (or its extract) is introduced to the sample pad, the sample moves through the interstitial space by capillary action to the conjugate pad that holds the detector agent. The target analyte, if present, will bind to the detector agent that is conjugated with a signal generator. The immunocomplexes will be anchored to the test line(s) where capture agents are coated on the detection pad. Accumulation of the signal generator at the test line(s) enables the immunocomplexes to be detected while unreacted substances continue to migrate to the absorbent pad.

### 2.2. Components of LFI

#### 2.2.1. Sample Pad

As the first component of the LFI to encounter the sample, the sample pad plays a crucial role in flow regulation because flooding of the device will adversely affect the assay performance. Woven meshes or cellulose fiber is typically used but the sample pad material can be suited for specific tasks such as the FUSION 5 membrane (Whatman, Kent, UK) that can act as an effective blood separator and prevent red blood cells from obscuring the signal generated on the detection pad [16]. Additionally, modifying agents such as proteins, viscosity enhancers, surfactants and/or buffer salts can be introduced at the sample pad to promote optimal test conditions. Different modifying agents may be used to serve different purposes such as to block the detection pad, to alter the sample viscosity, to aid in the rehydration and release of signal generator and to facilitate antigen–antibody binding by altering the chemical nature of the sample [17].

#### 2.2.2. Conjugate Pad

The conjugate pad is made of a porous material, such as glass fiber filter, to provide a matrix onto which desiccated signal generator can be held in a stable and functional state. The signal generator is functionalized with detector agents, allowing the formation of complete immunocomplexes to be detected visually or measured using a benchtop/handheld optical reader. Colloidal gold is usually employed as the signal generator in LFIs because it is relatively inexpensive, non-toxic, easy to synthesize and functionalize, stable in both liquid and dried forms, can be visualized with the naked eye and does not succumb to photodecomposition [16,18,19]. Functionality of the conjugates is preserved in the desiccated state with the help of a stabilizer such as sucrose or trehalose sugar. These water substitutes maintain the structural integrity of dried proteins through hydrogen bonding [20]. The amount of analyte in a sample that can be detected by a lateral flow strip is dictated by the volume of sample that is needed to release all the rehydrated signal generators from the conjugate pad because the subsequent formation of incomplete immunocomplexes would not contribute to the signal generation on the detection pad. A pink to red colored signal is generally obtained with gold conjugates but signal generation in other colors can also be achieved such as by using colored latex particles. Other signal generators such as carbon, selenium, liposomes, chemiluminescent and fluorescent nanoparticles are used less often [21]. In the case of fluorescent nanoparticles, a fluorescence analyzer is required for analysis, but the automated result interpretation eliminates the potential bias arising from the subjective judgement of the operator.

#### 2.2.3. Detection Pad

The detection pad represents one of the most important components of a LFI because reactions that occurred on this pad form the basis for result interpretation. Various materials that differ in pore size, porosity, thickness, and structural characteristics can be used as the detection pad including nylon, polyethersulfone, polyethylene, and glass fiber but nitrocellulose is still the material of choice because proteins interact and bind readily to the hydrophobic, neutral membrane via electrostatic interactions [21]. Nitrocellulose membranes with different capillary flow rates are commercially available but LFI developers would need to strike a balance between speed, cost, and assay performance as the sensitivity and total assay time decreases while specificity and reagent consumption increases with increasing capillary flow rate [17].

The test line is always dedicated towards the capture of the target analyte and a control line located downstream of the test line is also an integral part of all LFIs because it serves as built-in functionality and operational controls for each device. The control line ensures the reliability of the assay as multiple factors can negatively impact the assay performance including characteristic and composition of the sample, temperature variations during transport and storage, the use of faulty lateral flow devices or reagents, and improper execution of the assay procedure. The control line can be constructed with species-specific anti-Ig antibodies if the antibodies raised by the host are conjugated to the signal generator or with antibodies against a particular antigen if the said antigen is conjugated to the signal generator. However, the majority of FDA-EUA LFIs incorporate another set of signal generators that are conjugated with other antibodies (such as anti-biotin antibody, rabbit IgG and chicken IgY) or small molecules (such as dinitrophenyl) instead of the detector agent. This particular set of signal generators will only be captured at the control line in order to serve as a procedure control. Although such a control line would not be able to rule out false negative result due to dysfunctional detector agent–signal generator conjugates, factors influencing the development and validation of novel LFIs, such as cost, time and availability of supplies, during this public health crisis would need to be taken into consideration. Regardless of the target analyte which may or may not be present, a signal must be obtained at the control line. If the control line does not appear, the LFI result will be considered invalid and the sample has to be retested with a new lateral flow device.

#### 2.2.4. Absorbent Pad

The absorbent pad acts as a sink to accumulate unreacted substances including excess sample and reagents. Typically made of cellulose fibers, the absorbent pad drives the capillary action and must be able to accommodate the sample and buffer volume that are needed to perform the assay.

#### 2.2.5. Backing Card and Cassette

Proper lamination of the LFI materials on an adhesive backing card is a key factor in achieving consistent and uniform flow front. The backing card provides tensile strength and structural support for handling purposes but errors during the lamination process can lead to an irregular flow front and may even halt capillary flow. LFIs tend to be housed in a plastic cassette with internal pressure bars or pins to hold the device in position and to protect it from physical damage. Despite the additional cost incurred to house the strip within a cassette, there are several advantages to be gained. For example, the location of the sample loading site, the test line and the control line can be indicated on the cassette in order to facilitate proper assay execution and accurate result interpretation.

## 3. LFIs for SARS-CoV-2 Antigen Detection

### 3.1. Assay Format

SARS-CoV-2 has four major structural proteins: the spike (S), membrane (M), and envelope (E) that constitute the surface proteins and the nucleocapsid (N) protein that forms the ribonucleoprotein core inside the viral envelope. Among the 44 FDA-EUA antigen-detecting LFIs, 42 are directed against the N antigen whereas only two target both N antigen and receptor binding domain (RBD) of the S protein (Table 2). Almost half (47.7%) of the FDA-EUA antigen-detecting LFIs are over-the-counter, fully at-home diagnostic tests for COVID-19 [22] that can be purchased, conducted and interpreted at home without the involvement of a healthcare provider or a laboratory. The remaining LFIs are authorized to be used at patient care settings operating under a CLIA Certificate of Waiver as well as in CLIA-certified, high- or moderate-complexity laboratories. Notably, only two are multiplexed antigen tests namely Sofia 2 Flu + SARS Antigen FIA (Quidel) and Status COVID-19/Flu (Princeton BioMeditech Corporation, NJ, USA) that simultaneous detect and differentiate between SARS-CoV-2, influenza A and influenza B.

Monoclonal antibodies are prized for their high specificity and in the context of SARS-CoV-2 testing, the incidence of false-positive results and cross-reactivity with structurally similar antigens will be less likely as compared to polyclonal antibodies. Monoclonal antibodies in LFI are easier to optimize due to the homogeneity of the molecular species. However, the production process is not only tedious and costly but also requires technical expertise and specialized facilities. Unlike monoclonal antibodies, polyclonal antibodies are cheaper, quicker, and easier to produce but different batches of polyclonal antibodies will vary in quantity and quality. Polyclonal antibodies targeting different epitopes may capture the antigen more effectively, but assay performance can be compromised by the presence of non-specific antibodies and proteins. Heterogeneity of the polyclonal composition can complicate LFI optimization process as each Ig class and isotype may require slightly different conditions for binding and conjugation. Furthermore, potential structural and steric problems can arise from species such as IgA and IgM [16].

### 3.2. Specimen Type

The FDA-EUA LFIs for the detection of SARS-CoV-2 antigen are recommended to be performed within 5 to 12 days of symptom onset. The indicated specimen types for these antigen tests are currently limited to nasal swab and/or nasopharyngeal swab whereas a much wider range of specimens from the upper and lower respiratory tract can be tested with most of the FDA-EUA NAATs [8]. Nasal swab is the only acceptable specimen type in more than three-quarters of the FDA-EUA LFIs, followed by nasal and nasopharyngeal swabs in seven tests and nasopharyngeal swabs only in two of the tests. Unlike a nasopharyngeal specimen that requires a trained healthcare provider to collect, nasal specimens (anterior nares and mid-tubinate swabs) can be self-collected at the testing site or at home [23] with the procedure being less invasive and pain-free. Nonetheless, improper specimen collection and handling procedures can still adversely affect the test performance. For example, swabbing the nostril too quickly, swabbing only one of the nostrils and simply twirling or leaving the swab in the nose for several seconds may result in insufficient specimen for viral antigen detection [24].

The studies by Mak and colleagues highlighted the impacts on LFI sensitivity when different specimen types, including those that were outside of the manufacturer’s instructions for use (IFU), were tested in combination or alone [25,26]. In one of the studies, the highest sensitivity was obtained with nasopharyngeal and throat swabs (45.7%), followed by throat saliva (40%), nasopharyngeal aspirate and throat swab (34.3%) and sputum (11%) [26]. Given that the characteristics of these specimens differed substantially from one to another, the testing of specimen types outside of the manufacturer’s IFU may result in over dilution or a sub-optimal condition for antigen–antibody binding due to the changes in the chemical nature, matrix and viscosity of the specimen. In addition to the respiratory tract specimens, the detection of SARS-CoV-2 antigen in serum (n = 11/13) and urine specimen (*n* = 14/19) of COVID-19 cases with LFI have been reported in two separate preprints [27,28].

### 3.3. Diagnostic Marker

Unlike NAAT, LFI only detects antigens that are originally present in the specimen. Hence, the selection of target antigen is important as it relates directly to the test performance. Similar to SARS-CoV antigen detection [29,30], the N protein is the preferred target in FDA-EUA SARS-CoV-2 antigen-detecting LFIs because of its relative abundance during active infection [31]. Based on the clinical performance of several FDA-EUA antigen-detecting LFIs, the N protein can be detected as early as one day after symptom onset with specimen positivity ranging from 11.1% (n = 4/36) [32] to 43.8% (n = 14/32) [33]. Furthermore, positive correlations (r^2^ = 0.66–0.90) between SARS-CoV-2 N protein levels and RT-PCR Ct values in nasopharyngeal specimens have been reported in several studies [34,35,36].

The N protein is 419-amino acid in length and contains three distinct domains that interact with the viral genomic RNA via positively charged amino acid residues: the N-terminal domain (NTD; 46–176 residues), a serine/arginine-rich domain (SR-rich; 184–204 residues) in the linkage region and the C-terminal domain (CTD; 247–364 residues) [37,38]. With a size of 46 kDa, the N protein can be detected with the double antibody sandwich format. However, cross-reactivity between SARS-CoV-2 and SARS-CoV is frequently reported due to the high level of identity in the whole amino acid sequence (90.5%) [37] and epitope region sequence (78–100%) of the N protein [39]. Hence, positive results of FDA-EUA N antigen-detecting LFIs do not differentiate between SARS-CoV and SARS-CoV-2. Amino acid homology of the N protein between SARS-CoV-2 and other human coronaviruses shared a much lesser degree of identity that ranged from 28% (HCoV-229E) to 49% (MERS-CoV) [40].

### 3.4. Performance

In general, all the reagents and materials that are needed to perform the test are provided with the exception of a timer. A two-step procedure is typically adopted wherein a swab specimen is placed into the assay extraction buffer or reagent before the solution is introduced into the sample well of the lateral flow device followed by visual interpretation of the result within 10 to 30 min. While most of the lateral flow devices consist of a rectangular plastic cassette that houses the strip, the BinaxNOW COVID-19 Ag Card differs by housing the strip in a cardboard, book-shaped hinged test card. After the extraction reagent has been loaded and the swab specimen inserted into card, the extracted sample only comes into contact with the strip when the card is closed. The test results are categorized as either positive when both test and control lines appear, negative when only the control line appears or invalid when the control line does not appear. A positive result indicates the presence of SARS-CoV-2 antigens and confirmatory NAAT testing may be warranted based on the tested individual’s clinical and epidemiological characteristics. Negative results for individuals showing clinical signs and symptoms that are consistent with COVID-19 or with symptom onset beyond five days should be treated as presumptive and confirmed with a FDA-authorized NAAT.

For LFI that generates non-visually interpreted results, such as the Sofia SARS Antigen FIA (Quidel Corporation, San Diego, CA, USA), Sofia 2 Flu + SARS Antigen FIA (Quidel Corporation, San Diego, CA, USA), BD Veritor System for Rapid Detection of SARS-CoV-2 (BD) and Clip COVID Rapid Antigen Test (Luminostics, Fremont, CA, USA), the additional reader or analyzer required will entail a higher cost. However, these tests are generally more sensitive than naked eye detection and automation of the signal measurement permit additional analysis to be performed such as to correct for non-specific binding and to set the threshold to be applied for result interpretation. Automation of the result interpretation also reduces variability and eliminates the potential bias arising from the subjective judgement of the operator. Some analyzers, such as the BD Veritor Plus Analyzer (BD) and Sofia/Sofia2 (Quidel), offer the options of analyzing the LFI device after the test development has been timed manually (Analyze Now mode) or automated test development timing and analysis (Walk Away mode) by inserting the device immediately into the analyzer after sample application. The Ellume COVID-19 Home Test (Ellume) is the only FDA-EUA antigen-detecting LFI that integrates the analyzer (an optoelectronics reader system) within the housing of the lateral flow strip. In addition to the reagents and materials provided, the user must have a smartphone to download the Ellume COVID-19 Test App in order to connect with the LFI device. The user is also guided by a self-paced, step-by-step instructions to perform the test and the result will be automatically sent to the user’s smartphone and displayed via the downloaded application.

**Figure 2:**
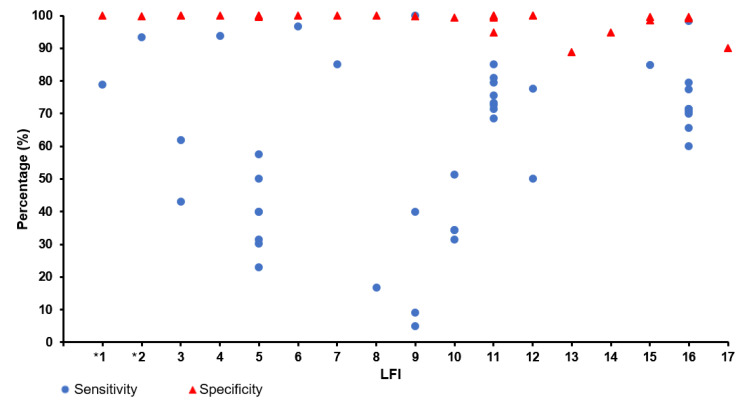
Sensitivity and specificity of commercial LFIs for the detection of SARS-CoV-2 antigen. (**1**). BD Veritor System for Rapid Detection of SARS-CoV-2 (VRD) (Becton, Dickinson and Company, Bergen, NJ, USA); (**2**). BinaxNOW COVID-19 Ag Card (Abbott Diagnostics, Chichago, IL, USA), (**3**). Biocredit COVID-19 Ag Detection Kit (RapiGEN, Anyang, South Korea); (**4**). Bioeasy 2019-Novel Coronavirus (2019-nCoV) Fluorescence Antigen Rapid Test Kit (fluorescence immunochromatographic assay) (Bioeasy Biotechnology, Shenzhen, China); (**5**). COVID-19 Ag Respi-Strip (Coris Bioconcept, Gembloux, Belgium); (**6**). COVID-VIRO (AAZ, Boulogne Billancourt, France); (**7**). Diagnostic Kit for 2019-nCoV Ag Test (Bioeasy Biotechnology, Shenzhen, China); (**8**). Huaketai New Coronavirus (Savant Biotechnology, Beijing, China); (**9**). Innova Lateral Flow Device (Innova Medical Group, Pasadena, CA, USA); (**10**). NADAL COVID-19 Ag Test (Nal Von Minden GmbH, Moers, Germany); (**11**). Panbio COVID-19 Ag Rapid Test Device (Abbott Rapid Diagnostics, Cologne, Germany); (**12**). Rapid antigen test provided by R-Biopharm; (**13**). Rapid COVID-19 Antigen Test (Healgen, Houston, TX, USA); (**14**). RIDA QUICK SARS-CoV-2 Antigen (R-Biopharm, Darmstadt, Germany); (**15**). SD Biosensor SARS-CoV-2 Rapid Antigen Test (Roche Diagnostics, Basel, Switzerland); (**16**). STANDARD Q COVID-19 Ag (SD Biosensor, Suwon-si, Republic of Korea); (**17**). StrongStep COVID-19 Antigen Test (Liming Bio-Products, Nanjing, China). * FDA-EUA.

Compared to the chemically labile RNA that is prone to degradation, viral antigen is a more robust analyte for SARS-CoV-2 testing and the use of LFI obviates tedious liquid-handling steps such as RNA extraction and amplification reaction mixture preparation. This simplicity in operation, however, comes at the expense of sensitivity. At present, the limit of detection (LoD) attained by FDA-EUA LFIs for SARS-CoV-2 antigen testing ranged from 28 to 50,000 TCID_50_/mL whereas the LoD of NAAT is generally well below 1 TCID_50_/mL [8]. The implication of selecting a LFI with a high LoD is an increased risk of misdiagnosis as the test would generate a false-negative test result when the level of antigen in the sample is below the LoD. Among the FDA-EUA LFIs, only the performance characteristics of BD Veritor System for Rapid Detection of SARS-CoV-2 and BinaxNOW COVID-19 Ag Card have been evaluated in peer and/or non-peer reviewed studies [41,42,43]. While both LFIs showed high specificity (99.9–100%), the sensitivity of BD Veritor System for Rapid Detection of SARS-CoV-2 (94.1%; 95% CI: 71.1–100%) was found to be higher than that of the BinaxNOW COVID-19 Ag Card (93.4%; 95% CI: 68.1–99.8%) [42,43]. In an attempt to compare the analytical sensitivity of BinaxNOW COVID-19 Ag Card to RT-PCR in terms of viral RNA copies, Perchetti and colleagues established that the LoD of the LFI was equivalent to 4.04–8.06 × 10^4^ copies/swab which corresponded to a C_T_ value of approximately 30 [41]. However, this LoD was determined with contrived specimens stored in phosphate-buffered saline and, hence, may not reflect the real life sensitivity of the assay with direct nasal swab. The comparison of sensitivity and specificity of various commercial LFIs for the detection SARS-CoV-2 antigen is presented in Figure 2, with the details available in Supplementary Table S1. The performance varied greatly between the LFIs with some assays demonstrating sensitivity below 50% but the specificity for all the LFIs ranged from 88.9 to 100%.

## 4. LFIs for the Detection of Anti-SARS-CoV-2 Antibodies

### 4.1. Assay Format

Serological LFIs that detect the presence of specific antibodies, such as IgM and IgG, against SARS-CoV-2 provide an indirect proof of COVID-19 infection as the individual has mounted an adaptive immune response to the infection. Although serological LFI is unsuitable to be an early diagnostic tool because it lags behind the molecular detection of viral genome, evidence of antibody seroconversion can be used to supplement the result of RT-PCR, to predict disease outcome, to identify eligible COVID-19 convalescent plasma donors as well as for epidemiological investigation and surveillance purposes [44,45,46]. The presence of IgM and IgG can be distinguished in a single lateral flow strip by constructing two test lines: one with anti-human IgM and another one with anti-human IgG. A selection of SARS-CoV-2 antigens such as N, S and fragments of the S protein (S1 subunit and RBD), which can be used individually or in combination, serve as the detector agent(s) that will be conjugated to a signal generator. Therefore, the target antibody would be sandwiched between the anti-human IgM/IgG and the SARS-CoV-2 antigen that is coupled to a signal generator (Figure 1c). A deviation from this format is seen in the CareStart COVID-19 IgM/IgG [47] wherein anti-SARS-CoV-2 IgM, if present, would form an immunocomplex with biotinylated anti-human IgM and SARS-CoV-2 antigens conjugated to colored particles. The biotinylated immunocomplex will be captured at the test line that is coated with streptavidin via affinity binding between biotin and streptavidin (Figure 1d).

Of the 20 FDA-EUA LFIs that detect IgM and/or IgG against SARS-CoV-2, only three LFIs were designed for the sole detection of IgG (RapCov Rapid COVID-19 Test [48], SCoV Detect IgG Rapid Test [49] and SGTi-flex COVID-19 IgG [50]) whereas the rest incorporate the detection of both IgM and IgG (Table 3). On the other hand, LFIs that are designed to detect total antibody against SARS-CoV-2 do not seek to differentiate between the different classes of Ig. At the time of writing, only one LFI has been granted FDA-EUA status for the detection of total antibody against SARS-CoV-2. The WANTAI SARS-CoV-2 Ab Rapid Test [51] uses the double-antigen sandwich format wherein RBD of the S protein is employed as both capture and detector agents. Antibodies that bind to the antigens coated on the test line and to the gold conjugates will result in a signal generation (Figure 1e).

### 4.2. Specimen Type

The types of specimens indicated for use with the FDA-EUA serological LFIs include venous whole blood, serum, plasma, oral fluid and/or finger-prick whole blood. While the time of specimen collection is not stated in the IFU of most FDA-EUA serological LFIs, there are some LFIs that are only intended for specimens that were collected after a specific period of time following the onset of symptoms and these time periods ranged from >7 days [52,53] to >14 days [54] after symptom onset. Among the indicated specimens, fingerprick whole blood is the easiest to be obtained without the need for a phlebotomist but only 11 serological LFIs are designed to accept fingerprick whole blood at the time of writing. The fingerprick whole blood has to be tested immediately, whereas the remaining abovementioned specimen types may be stored for a given duration at appropriate temperatures. Of note, only one serological LFI [55] is designed to work with oral fluid (gingival crevicular fluid). On the other hand, venous whole blood is an acceptable specimen in more than half of the FDA-EUA serological LFIs and it is collected using standard phlebotomy protocols into a blood collection tube containing anticoagulant such as sodium citrate, sodium heparin, and dipotassium EDTA. Since whole blood is only recommended to be stored at 2 to 8 °C for 2 to 3 days and not frozen for prolonged storage, the immediate collection of plasma is required if the test could not be run within 2 to 3 days of collection. The plasma can then be stored frozen at −20 °C or lower for one month.

### 4.3. Serological Marker

Given that IgM is generally the first class of Ig to be produced in response to an infection before class-switching to IgG, the presence of SARS-CoV-2-specific IgM provides an indication that the tested individual is at the early stage of infection. Nevertheless, IgM seroconversion occurring later than that of IgG and synchronous seroconversion of IgG and IgM among COVID-19 patients have also been observed [56]. The pentameric IgM has ten antigen-binding sites that contribute to its higher avidity towards antigen although the affinity is lower than that of IgG [57]. In contrast, IgG has two antigen-binding sites, exhibits higher specificity than IgM and is usually detectable after years of infection. In addition to IgM and IgG, IgA is also a potential marker for serological identification of SARS-CoV-2 infection. The kinetics of IgA was investigated in a longitudinal study of 19 COVID-19 patients and IgA levels were found to be consistently higher and persisted longer than IgM [46].

The immune response towards SARS-CoV-2 is generally similar to that of SARS-CoV although significant time dependences have been observed between the two viruses. After infection with SARS-CoV-2, IgM level peaks around 14 days post-symptom onset followed by a rapid declination in the third week whereas with SARS-CoV, the IgM peaks around three weeks post-symptom onset [58,59]. SARS-CoV-specific IgG also peaked later (around the fifth week of post-symptom onset) as compared to SARS-CoV-2-specific IgG that peaks around the second or third week post-symptom onset and remained high up to the fifth week [58,59]. In a study involving 285 COVID-19 patients, the median day of seroconversion for both IgG and IgM was 13 days post-symptom onset with 100% IgG seroconversion and 94.1% IgM seroconversion observed within 19 and 22 days post-symptom onset, respectively [56].

The N and S proteins of SARS-CoV-2 are known to be highly immunogenic and the main targets for antibody responses. A profiling study of anti-SARS-CoV-2 antibody seroconversion against the N protein revealed that IgM, IgA and IgG levels increased gradually within 1 to 3 weeks post-symptom onset with IgM and IgA peaking in the second and first week, respectively, whereas IgG continued to increase before reaching a plateau in the third week [60]. Notably, IgM, IgA and IgG could be detected as early as day 1 post-symptom onset, but the median time of appearance of IgM and IgA was at day 5 while for IgG was at day 14 [60]. A similar anti-SARS-CoV-2 antibody profile against the N and S proteins was described in another study. IgM and IgG were observed to share a similar dynamic pattern and level in the first two weeks post-symptom onset before IgG level continued to increase and surpassed that of IgM in the third week [61]. A meta-analysis has showed that the combined detection of IgG and IgM in LFIs resulted in greater sensitivity (78–83%) as compared to IgM and IgG alone (53–66%) but remained lower than those of ELISA- and chemiluminescent immunoassay-based tests (90–96%) [62]. In a separate study, greater sensitivity was attained by detecting total antibody against SARS-CoV-2 as compared to the detection of IgM and IgG alone or in combination [63]. However, less information may be derived from assays that do not distinguish the classes of Ig that were detected.

A previous study on SARS patients found that the antibody response was frequently and predominantly directed to the N protein instead of the S protein [64]. Compared to antibodies against other viral components (S, E and M proteins), anti-N antibodies were found to be more persistent and occurred in greater abundance [65]. Results from the study by Burbelo et al. [66] indicated that the detection of anti-N antibodies may be more sensitive for early identification of the infection as compared to those of the S protein counterparts, although contrasting results were reported in other studies [67,68]. The combined detection of N and S proteins by their IgM and IgG can potentially increase the SARS-CoV-2 detection rate in early infections [61]. Although cross-reactivity of anti-N and anti-S antibodies between SARS-CoV and SARS-CoV-2 have been reported in multiple studies [40,56,60,69] due to the high level of shared amino acid sequence identity (N, 90%; S, 77%), false-positive results due to cross-reactivity of SARS-CoV-specific antibodies has been postulated to be unlikely given that there were no SARS outbreaks since 2003 and as such, SARS-CoV-specific antibodies are unlikely to be present in the population [40].

### 4.4. Performance

The performance of some FDA-EUA LFIs in detecting anti-SARS-CoV-2 antibodies has been evaluated in several studies. The sensitivity of Healgen COVID-19 IgG/IgM Rapid Test Cassette was found to range from 67.7 to 100% with a specificity of 99% [70,71,72] whereas Biohit SARS-CoV-2 IgM/IgG Antibody Test Kit had a sensitivity and specificity of 94.6% (95% CI: 87.8–100%) and 92.6% (95% CI: 84.7–100%), respectively [73]. In another study, the WANTAI SARS-CoV-2 Ab Rapid Test was found to have a sensitivity and specificity of 83.1% (95% CI: 72.0–90.5%) and 98.0% (95% CI: 94.7–99.4%), respectively [70]. Greater variations in performance were observed for Innovita SARS-CoV-2 IgG/IgM antibody test kit whereby sensitivity and specificity of the assay ranged from 50 to 93% and 49 to 91%, respectively [74,75,76]. The comparison of sensitivity and specificity of various commercial LFIs for the detection of anti-SARS-CoV-2 antibodies is presented in Supplementary Figure S1 with the details available in Supplementary Table S2. Similar to antigen-detecting LFIs, the sensitivity of these serological LFIs varied greatly but specificity was more than 90% for most of the serological LFIs.

## 5. Conclusions and Future Perspective

As COVID-19 transitions from pandemic to endemic, the emergence of new variants of SARS-CoV-2 is expected [77]. Despite the significant progress that has been achieved in the development of medication, vaccines and diagnostics in the fight against COVID-19, new variants with increased transmissibility and/or disease severity can lead to a rapid surge in cases and quickly strain the finite healthcare resources. Accurate and rapid diagnosis continue to a crucial component to control COVID-19 outbreaks and to minimize the detrimental impacts on the healthcare system and economy. Although nucleic acid-based RT-PCR remained as the gold-standard diagnostic test, wide adoption and implementation of this technically intricate and equipment-dependent test are greatly hindered in countries and/or regions with weak or scarce laboratory infrastructure. Hence, the LFI platform provides an avenue to increase the capacity of COVID-19 testing and, more importantly, the rapid test can be deployed in both laboratory and non-laboratory settings and costs only a fraction of the price of RT-PCR test.

The availability of LFIs for the general population to perform self-testing at home has led to a tremendous increase in accessibility to COVID-19 testing while simultaneously allows appropriate containment measures to be taken early on to minimize the spread of COVID-19. Whereas LFI that detects SARS-CoV-2 antigen facilitate the diagnosis of infection early in its course, serological LFI can supplements nucleic acid-based diagnostics and assists in contact tracing, particularly among asymptomatic individuals. The feasibility of using LFI for SARS-CoV-2 detection by self-testing at home has been investigated in a large-scale study involving 1022 participants. The finding that 96% and 97% of the participants were able to perform the test without supervision and obtained a valid test result, respectively, advocates the suitability of LFI for mass self-testing [78]. The many advantages of LFI, which appeal to both end-users and manufacturers, make it a tool of choice in the field of point-of-care diagnostics but the platform is not without its own drawbacks. A major disadvantage of LFI is the subjective interpretation of the test result whereby ambiguity may arise in the interpretation of weak positive result even if the end users were skilled. A dedicated reader that is capable of imaging and quantifying the LFI result can be used to overcome operator bias [79] but this may lead to a substantial increase in the assay cost. Recent efforts to circumvent the need for a specialized readout device for LFI have focused on repurposing the smartphone as a reader by leveraging the phone’s built-in camera and capability to run mobile applications [80]. Furthermore, the smartphone also holds the potential for a test result to be uploaded in real-time to health-care related information systems to facilitate rapid reporting or for electronic record keeping purposes, but an active internet connection would become a requirement. The Ellume COVID-19 Home Test represents one such example, wherein the test incorporates the use of a smartphone (but not its camera) and a mobile application (Ellume COVID-19 Test App) to connect with the analyzer that is housed within the lateral flow device.

Another challenge associated with LFI lies in the improvement of the sensitivity and specificity of the assay. Some of the strategies that have been proposed focused on replacing colloidal gold as the signal generator and these include the use of lanthanide-doped polystyrene nanoparticles [79,81], multi-functional nanocomposite with a combination of magnetic-adhesion-color-nanozyme properties [82], and composite polymer beads that capitalize on two-wavelength imaging [83]. A nanoelectrokinetic-based sample enrichment step prior to the LFI [84] was also recently described but all these methods entailed the use of additional devices. Other strategies that have successfully increased the sensitivity of LFI without compromising the simplicity and practically of the platform were directed towards promoting the formation of the immune-complex on the lateral flow strip itself. For example, the addition of a macromolecular crowding agent, such as Ficoll MW 400,000 and Ficoll MW 70,000, led to a 5–10-fold improvement of the signal on commercially available LFIs [85]. A soluble time-delay wax barrier that selectively and temporarily accumulate the target and label nanoparticles on top of the test line was reported to generate a 51.7-fold and 96% enhancement in sensitivity and signal, respectively. Enhancement in the sensitivity and LoD of the LFI may also be attained via manufacturing technique. The laser-direct write technique, which was used to dispense a liquid photopolymer at specific regions of the nitrocellulose membrane, followed by photopolymerization to create impermeable walls inside the volume of the membrane was demonstrated to improve sensitivity and LoD by 62 and 30 times, respectively, as compared to conventional LFI [86]. Although LFI is a relatively old technology, various modifications and strategies have been described to improve its performance especially for clinical applications [87]. The adoption of these advances may improve the robustness, reliability and performance of LFI as a diagnostic tool beyond the COVID-19 pandemic.

## Figures and Tables

**Table 1:** An overview of differences between RT-PCR, LFI for the detection of SARS-CoV-2 antigen, ELISA and serological LFI.

	RT-PCR	LFI for the Detection of SARS-CoV-2 Antigen	ELISA	Serological LFI
Detection Target	SARS-CoV-2 nucleic acid	SARS-CoV-2 antigen	Antibodies against SARS-CoV-2	Antibodies against SARS-CoV-2
Sample Type	Upper and lower respiratory specimens	NS and NP swabs	Serum, plasma, venous whole blood	Serum, plasma, venous whole blood, finger-prick whole blood
Sensitivity	High	Moderate	High	Moderate
Specificity	High	High	High	High
Turnaround Time	2–3 h	10–15 min	2–5 h	10–15 min
Instrumentation	Required	Not required except for fluorescent LFI	Required	Not required
Test Complexity	Most are complex	Easy-to-use	Most are complex	Easy-to-use
Laboratory Requirements	High	Average	High	Average
Usage	To detect active infection	To detect active infection	To detect past infection	To detect past infection

NS, nasal; NP, nasopharyngeal.

**Table 2:** Characteristics of FDA-EUA LFIs for the detection of SARS-CoV-2 antigen.

Developer	Test Name	Diagnostic Marker(s)	Time-to-Result	Sample Indicated for Testing	LoD (TCID_50_/mL)	Authorized Setting(s)
InBios International Inc.	SCoV-2 Ag Detect Rapid Self-Test	N antigen	20–25 min	Anterior nasal (nares) swab samples	6.3 × 10^3^	Home, H, M, W
Quidel Corporation	QuickVue At-Home OTC COVID-19 Test	N antigen	10–15 min	Anterior nasal (nares) swab samples	1.91 × 10^4^	Home, H, M, W
ACON Laboratories, Inc.	Flowflex COVID-19 Antigen Home Test	N antigen	15–30 min	Anterior nasal (nares) swab samples	2.5 × 10^3^	Home, H, M, W
Xiamen Boson Biotech Co., Ltd.	Rapid SARS-CoV-2 Antigen Test Card	N antigen	15–30 min	Anterior nasal (nares) swab samples	1.4 × 10^2^	Home, H, M, W
Access Bio, Inc.	CareStart COVID-19 Antigen Home Test	N antigen	10–15 min	Anterior nasal (nares) swab samples	2.8 × 10^3^	Home, H, M, W
ANP Technologies, Inc.	NIDS COVID-19 Antigen Rapid Test Kit	N antigen	15–30 min	Mid-turbinate (MT) nasal swabs	3.11 × 10^2^	H, M, W
Genabio Diagnostics Inc.	Genabio COVID-19 Rapid Self-Test Kit	N antigen	15–20 min	Anterior nasal (nares) swab samples	1.78 × 10^4^	Home, H, M, W
OSANG LLC	OHC COVID-19 Antigen Self Test	N antigen	15–30 min	Anterior nasal (nares) swab samples	1.4 × 10^4^	Home, H, M, W
PHASE Scientific International, Ltd.	INDICAID COVID-19 Rapid Antigen At-Home Test	N antigen	20–25 min	Anterior nasal (nares) swab samples	2.8 × 10^3^	Home, H, M, W
PHASE Scientific International, Ltd.	INDICAID COVID-19 Rapid Antigen Test	N antigen	20–25 min	Anterior nasal (nares) swab samples	2.8 × 10^3^	H, M, W
Celltrion USA, Inc.	Celltrion DiaTrust COVID-19 Ag Rapid Test	N and RBD antigens	15–20 min	Mid-turbinate (MT) nasal swabs	3.2 × 10^1^	H, M, W
GenBody Inc.	GenBody COVID-19 Ag	N antigen	15–20 min	Nasopharyngeal (NP) or anterior nasal (AN) swab specimens	1.11 × 10^2^	H, M, W
Siemens Healthineers	CLINITEST Rapid COVID-19 Antigen Self-Test	N antigen	15–20 min	Anterior nasal (nares) swab samples	7.0 × 10^3^	Home, H, M, W
OraSure Technologies, Inc.	InteliSwab COVID-19 Rapid Test Rx	N antigen	30–40 min	Anterior nasal (nares) swab samples	2.5 × 10^2^	Home, H, M, W
OraSure Technologies, Inc.	InteliSwab COVID-19 Rapid Test	N antigen	30–40 min	Anterior nasal (nares) swab samples	2.5 × 10^2^	Home, H, M, W
OraSure Technologies, Inc.	InteliSwab COVID-19 Rapid Test Pro	N antigen	30–40 min	Anterior nasal (nares) swab samples	2.5 × 10^2^	H, M, W
Maxim Biomedical, Inc.	MaximBio ClearDetect COVID-19 Antigen Home Test	N antigen	15–30 min	Anterior nasal (nares) swab samples	7.5 × 10^2^	Home, H, M, W
SD Biosensor, Inc.	Pilot COVID-19 At-Home Test	N antigen	20–30 min	Anterior nasal (nares) swab samples	1.4 × 10^3^	Home, H, M, W
Ellume Limited	Ellume COVID-19 Home Test	N antigen	15 min	Mid-turbinate (MT) nasal swabs	6.31 × 10^3^	Home, H, M, W
iHealth Labs, Inc.	iHealth COVID-19 Antigen Rapid Test	N antigen	15–30 min	Anterior nasal (nares) swab samples	20 × 10^3^	Home, H, M, W
Watmind USA	Speedy Swab Rapid COVID-19 Antigen Self-Test	N antigen	15–30 min	Anterior nasal (nares) swab samples	2.8 × 10^2^	Home, H, M, W
Celltrion USA, Inc.	Celltrion DiaTrust COVID-19 Ag Home Test	N and RBD antigens	15–20 min	Mid-turbinate (MT) nasal swabs	2.8 × 10^1^	Home, H, M, W
Salofa Oy	Sienna-Clarity COVID-19 Antigen Rapid Test Cassette	N antigen	10–20 min	Nasopharyngeal (NP) swab specimens	1.25 × 10^3^	H, M, W
QIAGEN GmbH	QIAreach SARS-CoV-2 Antigen	N antigen	2–15 min	Nasopharyngeal (NP) or anterior nasal (AN) swab specimens	5 × 10^4^	H, M
InBios International, Inc.	SCoV-2 Ag Detect Rapid Test	N antigen	20–25 min	Anterior nasal (nares) swab samples	6.3 × 10^3^	H, M, W
Abbott Diagnostics Scarborough, Inc.	BinaxNOW COVID-19 Ag Card Home Test	N antigen	15 min	Anterior nasal (nares) swab samples	1.41 × 10^2^	Home, H, M, W
Abbott Diagnostics Scarborough, Inc.	BinaxNOW COVID-19 Antigen Self Test	N antigen	15–30 min	Anterior nasal (nares) swab samples	7 × 10^2^	Home, H, M, W
Oceanit Foundry LLC	ASSURE-100 Rapid COVID-19 Test	N antigen	20–30 min	Anterior nasal (nares) swab samples	1.41 × 10^2^	H, M, W
Becton, Dickinson and Company (BD)	BD Veritor At-Home COVID-19 Test	N antigen	15–20 min	Anterior nasal (nares) swab samples	0.88 × 10^2^	Home, H, M, W
Luminostics, Inc.	Clip COVID Rapid Antigen Test	N antigen	45 s	Anterior nasal (nares) swab samples	1.87 × 10^2^	H, M, W
Abbott Diagnostics Scarborough, Inc.	BinaxNOW COVID-19 Ag Card	N antigen	15–30 min	Anterior nasal (nares) swab samples	1.41 × 10^2^	H, M, W
Abbott Diagnostics Scarborough, Inc.	BinaxNOW COVID-19 Ag 2 Card	N antigen	15–30 min	Anterior nasal (nares) swab samples	1.41 × 10^2^	H, M, W
Nano-Ditech Corp.	Nano-Check COVID-19 Antigen Test	N antigen	15–20 min	Nasopharyngeal (NP) swab specimens	7 × 10^2^	H, M, W
iHealth Labs, Inc.	iHealth COVID-19 Antigen Rapid Test Pro	N antigen	15–30 min	Anterior nasal (nares) swab samples	20 × 10^3^	H, M, W
Becton, Dickinson and Company (BD)	BD Veritor System for Rapid Detection of SARS-CoV-2	N antigen	15–20 min	Anterior nasal (nares) swab samples	1.4 × 10^2^	H, M, W
Access Bio, Inc.	CareStart COVID-19 Antigen test	N antigen	10–15 min	Nasopharyngeal (NP) or anterior nasal (AN) swab specimens	8 × 10^2^	H, M, W
Quidel Corporation	QuickVue SARS Antigen Test	N antigen	10–15 min	Anterior nasal (nares) swab samples	7.57 × 10^3^	H, M, W
Princeton BioMeditech Corp.	Status COVID-19-Flu A&B	N antigen	15–20 min	Nasopharyngeal (NP) or anterior nasal (AN) swab specimens	2.7 × 10^3^	H, M, W
Xtrava Health	SPERA COVID-19 Ag Test	N antigen	15–30 min	Anterior nasal (nares) swab samples	1.56 × 10^3^	H, M, W
Ellume Limited	ellume.lab COVID Antigen Test	N antigen	3–15 min	Mid-turbinate (MT) nasal swabs	7.16 × 10^3^	H, M, W
Quidel Corporation	Sofia SARS Antigen FIA	N antigen	15–30 min	Anterior nasal (nares) swab samples	2.8 × 10^2^	H, M, W
Becton, Dickinson and Company (BD)	BD Veritor System for Rapid Detection of SARS-CoV-2 & Flu A + B	N antigen	15–20 min	Anterior nasal (nares) swab samples	1.13 × 10^2^	H, M, W
Quidel Corporation	QuickVue At-Home COVID-19 Test	N antigen	10–15 min	Anterior nasal (nares) swab samples	1.91 × 10^4^	Home, H, M, W
Quidel Corporation	Sofia 2 Flu + SARS Antigen FIA	N antigen	15–30 min	Nasopharyngeal (NP) or anterior nasal (AN) swab specimens	9.17 × 10^1^	H, M, W

**Table 3:** Characteristics of FDA-EUA serological LFIs for the detection of antibodies against SARS-CoV-2.

Developer	Test Name	Serological Marker	Antigenic Target(s)	Time-to-Result	Sample Indicated for Testing	Combined Sensitivity (95% CI)	Combined Specificity (95% CI)	Authorized Setting(s)
Innovita (Tangshan) Biological Technology Co., Ltd.	Innovita 2019-nCoV Ab Test (Colloidal Gold)	IgM and IgG	S1 and N	10–15 min	Serum, plasma, venous whole blood	93.3% (78.7–98.2%)	98.8% (93.3–99.8%)	H, M
Megna Health, Inc.	Rapid COVID-19 IgM/IgG Combo Test Kit	IgM and IgG	S1 and N	15–20 min	Serum, plasma, fingerprick whole blood	100% (88.7–100%)	95% (87.8–98%)	H, M, W
Jiangsu Well Biotech Co., Ltd.	Orawell IgM/IgG Rapid Test	IgM and IgG	RBD	10–15 min	Serum, plasma	100% (93.8–100%)	94.8% (88.5–97.8%)	H, M
QIAGEN, GmbH	QIAreach Anti-SARS-CoV-2 Total Test	Total Antibody	S1	10 min	Serum, plasma	100% (88.7–100%)	97.5% (91.3–99.3%)	H, M
Hangzhou Laihe Biotech Co., Ltd.	LYHER Novel Coronavirus (2019-nCoV) IgM/IgG Antibody Combo Test Kit (Colloidal Gold)	IgM and IgG	S1	10–15 min	Serum, plasma	100% (88.7–100%)	98.8% (93.3–99.8%)	H, M
NOWDiagnostics, Inc.	ADEXUSDx COVID-19 Test	Total Antibody	RBD	15–30 min	Serum, plasma, venous whole blood, fingerprick whole blood	93.3% (78.7–98.2%)	100% (95.4–100%)	H, M, W
Assure Tech. (Hangzhou Co., Ltd.)	Assure COVID-19 IgG/IgM Rapid Test Device	IgM and IgG	S1 and N	15–30 min	Serum, plasma, venous whole blood, fingerprick whole blood	100% (88.7–100%)	98.8% (93.3–98.8%)	H, M, W
Healgen Scientific LLC	COVID-19 IgG/IgM Rapid Test Cassette (Whole Blood/Serum/Plasma)	IgM and IgG	S1	10–15 min	Serum, plasma, venous whole blood	100% (88.7–100%)	97.5% (91.3–99.3%)	H, M
Biohit Healthcare (Hefei) Co. Ltd.	Biohit SARS-CoV-2 IgM/IgG Antibody Test Kit	IgM and IgG	N	15–20 min	Serum, plasma, venous whole blood	96.7% (83.3–99.4%)	95% (87.8–98%)	H, M
ACON Laboratories, Inc.	ACON SARS-CoV-2 IgG/IgM Rapid Test	IgM and IgG	-	15–20 min	Serum, plasma, venous whole blood	100% (88.7–100%)	97.5% (91.3–99.3%)	H, M
NanoEntek America, Inc.	FREND COVID-19 total Ab	Total Antibody	N	3–4 min	Plasma	96.7% (83.3–99.4%)	98.8% (93.3–99.8%)	H, M
Sugentech, Inc.	SGTi-flex COVID-19 IgG	IgG	N and RBD	10–30 min	Serum, plasma, venous whole blood, fingerprick whole blood	96.7% (83.3–99.4%)	100% (95.4–100%)	H, M, W
Nirmidas Biotech, Inc.	MidaSpot COVID-19 Antibody Combo Detection Kit	IgM and IgG	-	18–25 min	Serum, plasma, fingerprick whole blood	100% (88.7–100%)	96.2% (89.5–98.7%)	H, M, W
InBios International, Inc.	SCoV-2 Detect IgG Rapid Test	IgG	S	20–25 min	Serum, plasma, venous whole blood, fingerprick whole blood	100% (88.7–100%)	100% (95.4–100%)	H, M, W
Access Bio, Inc.	CareStart COVID-19 IgM/IgG	IgM and IgG	N and RBD	10–15 min	Serum, plasma, venous whole blood, fingerprick whole blood	100% (88.7–100%)	97.5% (91.3–99.3%)	H, M, W
Diabetomics, Inc.	CovAb SARS-CoV-2 Ab Test	Total Antibody	S1	15–20 min	Oral fluid (gingival crevicular fluid)	-	-	H, M, W
Salofa Oy	Sienna-Clarity COVIBLOCK COVID-19 IgG/IgM Rapid Test Cassette	IgM and IgG	RBD	10–20 min	Serum, plasma, venous whole blood, fingerprick whole blood	93.3% (78.7–98.2%)	98.8% (93.3–99.8%)	H, M, W
Access Bio, Inc.	CareStart EZ COVID-19 IgM/IgG	IgM and IgG	N and RBD	15–20 min	Serum, plasma, venous whole blood, fingerprick whole blood	100% (88.7–100%)	100% (95.4–100%)	H, M, W
ADVAITE, Inc.	RapCov Rapid COVID-19 Test	IgG	N	15–20 min	fingerprick whole blood	90% (73.6–97.3%)	95.2% (89.2–97.9%)	H, M, W
Beijing Wantai Biological Pharmacy Enterprise Co., Ltd.	WANTAI SARS-CoV-2 Ab Rapid Test	Total Antibody	RBD	15–20 min	Serum, plasma, venous whole blood	100% (88.7–100%)	98.8% (93.3–99.8%)	H, M
Hangzhou Biotest Biotech Co., Ltd.	RightSign COVID-19 IgG/IgM Rapid Test Cassette	IgM and IgG	RBD	10–20 min	Serum, plasma, venous whole blood, fingerprick whole blood	100% (88.7–100%)	100% (95.4–100%)	H, M, W
Xiamen Biotime Biotechnology Co., Ltd.	BIOTIME SARS-CoV-2 IgG/IgM Rapid Qualitative Test	IgM and IgG	-	20–30 min	Serum, plasma, venous whole blood	96.7% (83.3–99.4%)	97.5% (91.3–99.3%)	H, M
Nirmidas Biotech, Inc.	Nirmidas COVID-19 (SARS-CoV-2) IgM/IgG Antibody Detection Kit	IgM and IgG	S1 and RBD	10–15 min	Serum, plasma	-	-	H, M
TBG Biotechnology Corp.	TBG SARS-CoV-2 IgG/IgM Rapid Test Kit	IgM and IgG	S and N	15–20 min	Serum, plasma	93.3% (78.7–98.2%)	95% (87.8–98%)	H, M
Biocan Diagnostics Inc.	Tell Me Fast Novel Coronavirus (COVID-19) IgG/IgM Antibody Test	IgM and IgG	S and N	10–15 min	Serum, plasma, venous whole blood	100% (94.9–100%)	99.4% (96.6–99.9%)	H, M

## Supplementary Materials

The following supporting information can be downloaded at: https://www.mdpi.com/article/10.3390/diagnostics12112854/s1, Figure S1: Sensitivity and specificity of the commercial LFIs for the detection of antibody against SARS-CoV-2, Table S1: Comparison of performance evaluation between commercial LFIs for the detection of SARS-CoV-2 antigen [25,41,42,43,88,89,90,91,92,93,94,95,96,97,98,99,100,101,102,103,104,105,106,107,108,109,110,111,112,113], Table S2: Comparison of performance evaluation between commercial serology LFIs for the detection of antibody against SARS-CoV-2 [70,71,72,73,74,75,76,114,115,116,117,118,119,120,121,122,123,124,125,126,127,128,129,130,131].
