# Supplementary material for: Lateral Flow Immunoassays for SARS-CoV-2
Source: Diagnostics (Basel). 2022 Nov 18;12(11):2854. doi: 10.3390/diagnostics12112854 (PMC9689684; doi:10.3390/diagnostics12112854)
Supplement: Supplementary file 1 [file diagnostics-12-02854-s001.zip › Figure S1.pdf]

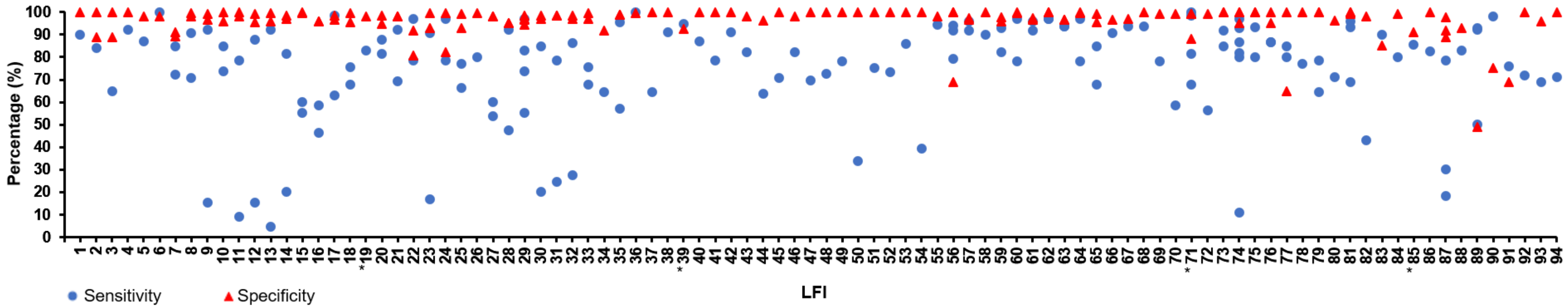

Figure S1. Sensitivity and specificity of the commercial LFIs for the detection of antibody against SARS-CoV-2. 1. BioMedomics COVID-19 IgG/IgM Rapid Antibody Test; 2. Biosynex COVID-19 BSS (Biosynex, Switzerland, Fribourg); 3. COVID-19 Sign IgM/IgG (Servibio/VEDALAB, France, Alençon); 4. NTBIO (NTBIO Diagnostics Inc., Surrey, British Columbia, Canada); 5. Orient-Gene (Zhejiang Orient-Gene Biotech Co. Ltd., Huzhou, China); 6. MEDsan (MEDsan GmbH, Biological Health Solutions, Hamburg, Germany); 7. iCare Covid-19 Rapid Test (Covid-19 IgG/IgM Rapid test Kit); 8. NADAL COVID-19 IgG/IgM Test; 9. BIOZEK Medical COVID-19 IgG/IgM Rapid Test Cassette; 10. BIOSYNEX COVID −19 BSS; 11. Panbio COVID-19 IgG/IgM Rapid Test Device; 12. Acro 2019-nCoV IgG/IgM Rapid Teset; 13. ichroma COVID-19 Ab+ ichroma II instrument; 14. COVID-19 IgG-IgM Rapid test; 15. Diagnostic Kit for IgM/IgG Antibody to Coronavirus (SARS-CoV-2) (Lateral Flow); 16. COVISURE™ COVID-19 IgG-IgM Rapid Test; 17. STANDARD Q COVID-19 IgM/IgG Combo Test; 18. Novel Coronavirus (2019-nCoV) IgG/IgM Test Kit (Colloidal gold); 19. WANTAI SARS-CoV-2 Ab Rapid Test; 20. Leccurate SARS-CoV-2 Antibody Test Kit; 21. OnSite Covid-19 IgG/IgM; 22. COVID-19 IgG/IgM Rapid Test Kit; 23. Anti-SARS-CoV-2 Rapid Test; 24. Instant-View COVID-19 IgG/IgM Antibody Test; 25. 2019-nCOV IgG/IgM rapid test; 26. INgezim COVID 19 CROM (kassett); 27. SARS -CoV-2 IgM/IgG Antibody Detection Kit; 28. COVID19 IgG & IgM Test Kit(colloidal gold method); 29. COVID-19 IgG/IgM Rapid Test; 30. 2019-nCovid IgG/IgM Rapid Test Cassette; 31. Diagnostic Kit for SARS-Cov-2 IgM/IgG Antibody (Collodial Gold); 32. nCOVID-19 IgG & IgM POCT; 33. StrongStep® COVID-19 IgG/IgM Combo Test; 34. EBS Alert SARS-CoV-2 ANTIBODY RAPID TEST; 35. Chembio DPP COVID-19 IgM/IgG System 2.0; 36. LumiraDx SARS-CoV-2 Ab Test; 37. API; 38. API (v2); 39. BioHit; 40. BTNX; 41. Camtech; 42. CareHealth; 43. Cellex; 44. Edinburgh; 45. Genobio; 46. InTec; 47. KHB; 48. Lumiquick; 49. Oranoxis; 50. OZO; 51. Phamatech; 52. Ray Biotech; 53. Ray Biotech (v2); 54. U2U; 55. Vivachek; 56. Biosensor; 57. AMS International; 58. Leccurate; 59. HIGHTOP One Step rapid test; 60. Cromatest COVID-19; 61. AMP Rapid Test; 62. Egens; 63. Cellex; 64. Onesite Rapidtest; 65. OnSite COVID-19 IgG/IgM Rapid Test (CTK Biotech Inc., CA, USA); 66. Hangzhou AllTest COVID-19 test (Hangzhou AllTest Biotech, Hangzhou, China); 67. Wondfo SARS-CoV-2 Antibody Test (Guangzhou Wondfo Biotech, Guagzhou, China); 68. Hightop SARS-CoV-2 IgM/IgG Antibody Rapid Test; 69. 2019-nCoV IgG/IgM Rapid Test (Acro Biotech Inc., CA, USA); 70. Anti-SARS-CoV-2 Rapid Test (Autobio Diagnostics Co. Ltd, Zhengzhou, China); 71. Healgen COVID-19 IgG/IgM Rapid Test Cassette; 72. NADAL COVID-19 IgG/IgM Test (Nal von Minden GmbH, Moers, Germany); 73. Accu-Tell COVID-19 IgG/IgM Rapid Test (AccuBioTech, Beijing, China); 74. The Diagnostic Kit for IgM / IgG Antibody to Coronavirus (SARS-CoV-2) (Lateral Flow) (Zhuhai Livzon Diagnostics, Zhuhai, China); 75. Coronavirus (COVID-19) IgM/IgG Rapid Test Kit (ISIA BIO-Technology, Chongqing, China); 76. H2019-nCoV IgM Antibody Test Kit (Hecin Biotech Co., Ltd., Guangzhou, China); 77. COVID-19 IgG/IgM Rapid Test Kit (UNscience Biotechnology, Wuhan, China); 78. 2019-nCoV IgM/IgG Rapid Test (Acro Biotech, CA, USA); 79. AllTest 2019-nCoV IgG/IgM Rapid Test Cassette (Hangzhou AllTest Biotech,

Hangzhou, China); 80. Colloidal gold immunochromatography antibody detection kit (Shanghai Outdo Biotech, China); 81. COVID-19 IgG/IgM Rapid Test Cassette (Zhejiang Orient Gene Biotech, Huzhou, China); 82. One Step Novel Coronavirus (COVID-19) IgM/IgG Antibody Test (Artron, Burnaby, Canada); 83. Rapid SARS -CoV-2 Antibody (IgM/IgG) Test (InTec, Xiamen, China); 84. qSARS-CoV-2 IgG/IgM Cassette Rapid Test (GICA) (Cellex, NC, USA); 85. SARS-CoV-2 IgG/IgM antibody test kit (Innovita Biological Technology Co., Ltd, Tangshan, Hebei, China); 86. The Diagnostic Kit for IgM / IgG Antibody to Coronavirus (SARS-CoV-2) (Lateral Flow) (Zhuhai Livzon Diagnostics, Zhuhai, China); 87. VivaDiag COVID-19 IgM/IgG Rapid Test (VivaChek, Wilmington, USA); 88. 2019-nCoV IgG/IgM Antibody Rapid Test Kit (Beijing Diagreat Biotechnologies, Beijing, China); 89. 2019-nCoV Ab Test (Colloidal Gold) (Innovita Biological Technology, Beijing, China); 90. Wondfo test (Guangzhou Wondfo Biotech, Guagzhou, China); 91. Runkun test (Runkun Pharmaceutical, Hunan, China); 92. 2019-n-CoV IgG/IgM rapid test cassette (Bio Marketing Diagnostics, Akiva, Israel); 93. Novel Coronavirus (2019-n-CoV) antibody IgG/IgM assay (colloidal gold) (Avioq, Bio-Tech, Shandong, China); 94. QuickZen COVID-19 IgM/IgG Kit (ZenTech, Angleur, Belgium). *FDA-EUA.
