# Supplementary material for: Lateral Flow Immunoassays for SARS-CoV-2
Source: Diagnostics (Basel). 2022 Nov 18;12(11):2854. doi: 10.3390/diagnostics12112854 (PMC9689684; doi:10.3390/diagnostics12112854)
Supplement: Supplementary file 1 [file diagnostics-12-02854-s001.zip › Table S1.pdf]

Table S1. Comparison of performance evaluation between commercial LFIs for the detection of SARS-CoV-2 antigen.

Test Name (Developer)	Main Findings	*N*	Subject Categorization	Sensitivity (%)	Specificity (%)	Reference
Innova SARS-CoV-2 antigen rapid lateral flow test (Innova Medical group, CA, US)	This assay is a valuable tool to identify asymptomatic individuals and those with high viral loads.	5869	5869 asymptomatic adults;	40	99.9	[1]
BD Veritor System for Rapid Detection of SARS-CoV-2 (VRD) (Becton, Dickinson and Company, New Jersey, US)*	This assay is a promising diagnostic test for community screening of symptomatic individuals during first week of symptom onset.	352	352 symptomatic adults; 123 RT-PCR positive	94.1	100	[2]
BinaxNOW COVID-19 Ag Card (Abbott Diagnostics, Chichago, US)*	This assay has an analytical sensitivity equivalent to that of a generic RT-PCR C_T of 29-30.	72	72 RT-PCR positive	NA	NA	[3]
BinaxNOW COVID-19 Ag Card (Abbott Diagnostics, Chichago, US)*	This assay accurate detected specimens with high viral loads in both symptomatic and asymptomatic individuals.	878	Ongoing community transmission; 26 RT-PCR positive	93.4	99.9	[4] †
Biocredit COVID-19 Ag Detection Kit (RapiGEN, Anyang, South Korea)	This assay has poor sensitivity and should not be used for diagnosis.	310	188 RT-PCR positive	43.1	NA	[5]
1. Panbio COVID-19 Ag Rapid Test Device (Abbott Rapid Diagnostics, Cologne, Germany);<br>2. Biocredit COVID-19 Ag Detection Kit (RapiGEN, Anyang, South Korea);<br>3. Rapid COVID-19 Antigen Test (Healgen, Houston, USA);<br>4. COVID-19 Ag Respi-Strip (Coris Bioconcept, Gembloux, Belgium);<br>5. RIDA QUICK SARS-CoV-2 Antigen (R-Biopharm, Darmstadt, Germany);<br>6. NADAL COVID-19 Ag Test (Nal Von Minden GmbH, Germany);<br>7. SD Biosensor SARS-CoV-2 Rapid Antigen Test (Roche Diagnostics, Basel, Switzerland)	Most of the assays have sensitivity that overlaps with viral load during first week of symptoms.	273	138 RT-PCR positive; 100 other respiratory pathogens; 35 healthy volunteers	NA	1. 99.3<br>2. 100<br>3. 88.9<br>4. 100<br>5. 94.8<br>6. 99.3<br>7. 98.5	[6]
1. Biocredit COVID-19 Ag Detection Kit (RapiGEN, Anyang, South Korea);<br>2. StrongStep COVID-19 Antigen Test (Liming Bio-Products, Nanjing, China);<br>3. Huaketai New Coronavirus (Savant Biotechnology, Beijing, China);	The test performance varied between different manufacturers.	111	111 symptomatic patients; 80 RT-PCR positive; 31 RT-PCR negative	1. 62<br>2. 0 (suspended)<br>3. 16.7<br>4. 85	1. 100<br>2. 90<br>3. 100<br>4. 100	[7]

4. Diagnostic Kit for 2019-nCoV Ag Test (Bioeasy Biotechnology, Shenzhen, China)						
Bioeasy 2019-Novel Coronavirus (2019-nCoV) Fluorescence Antigen Rapid Test Kit (fluorescence immunochromatographic assay) (Bioeasy Biotechnology, Shenzhen, China)	This assay showed high sensitivity and specificity in the first week of symptoms.	127	82 RT-PCR positive	93.9	100	[8]
COVID-19 Ag Respi-Strip (Coris Bioconcept, Gembloux, Belgium)	A diagnostic algorithm was proposed for the use of the assay to complement RT-PCR.	328	NA	57.6	99.5	[9]
COVID-19 Ag Respi-Strip (Coris Bioconcept, Gembloux, Belgium)	The assay was more sensitive for samples with high viral loads.	138	94 RT-PCR positive	50	100	[10]
COVID-19 Ag Respi-Strip (Coris Bioconcept, Gembloux, Belgium)	This assay has poor sensitivity and does not reduce the number of samples outsourced for confirmation by RT-PCR.	774	Out of 771 negative COVID-19 Ag Respi-Strip, 159 positive by RT-PCR	NA	NA	[11]
COVID-19 Ag Respi-Strip (Coris Bioconcept, Gembloux, Belgium)	This assay should not be used alone for diagnosis and it shows no benefit in reducing the use of RT-PCR.	148	106 RT-PCR	30.2	NA	[12]
1. COVID-19 Ag Respi-Strip (Coris Bioconcept, Gembloux, Belgium);<br>2. NADAL COVID-19 Ag Test (Nal Von Minden GmbH, Germany);<br>3. STANDARD Q COVID-19 Ag (SD Biosensor, Korea)	Coris and NADAL were 10^5 fold and STANDARD Q was 10^2 fold less sensitive than RT-PCR. Throat saliva is not recommended for test. Adoption of these assays for clinical use should be guided by evidences.	280	280 RT-PCR positive (35 throat saliva; 35 NP & throat swabs; 35 NP aspirate & throat swab; 35 NP swabs)	NP aspirate & throat swab: 22.9; 34.3; 60<br>NP swab & throat swab: 40; 34.3; 71.4<br>NP swab: 40; 51.4; 65.7<br>Throat saliva: 31.4; 31.4; 71.4	NA	[13]
COVID-VIRO (AAZ, Boulogne Billancourt, France)	This assay met the performance recommended by WHO.	248	121 RT-PCR positive; 127 RT-PCR negative	96.7	100	[14]
Innova Lateral Flow Device (Innova Medical group, CA, US)	This assay can detect sample with viral titre above ~100 viral copies/ml but incapable of detecting sample with $C_T \geq 30$.	7189	7189 asymptomatic students; 720 confirmed by RT-PCR (8 RT-PCR positive)	100 ($C_T < 29$); 9.1 ($C_T \leq 29$); 5.01 ($C_T < 33$)	NA	[15]
Panbio COVID-19 Ag Rapid Test Device (Abbott Rapid Diagnostics, Cologne, Germany)	This assay has the same LoD to that of STANDARD Q COVID-19 Ag.	105	105 RT-PCR positive (35 NP & throat swabs; 35 NP swab; 35 throat saliva)	68.6	NA	[16]

Panbio COVID-19 Ag Rapid Test Device (Abbott Rapid Diagnostics, Cologne, Germany)	The assay performed well for early diagnosis.	412	412 symptomatic patients; 43 RT-PCR positive	79.6	100	[17]
Panbio COVID-19 Ag Rapid Test Device (Abbott Rapid Diagnostics, Cologne, Germany)	This assay has high sensitivity and specificity during the first week of symptoms.	225	60 RT-PCR positive	73.3	NA	[18]
Panbio COVID-19 Ag Rapid Test Device (Abbott Rapid Diagnostics, Cologne, Germany)	This assay may be beneficial in community-based surveillance of symptomatic individuals.	1367 (Utrecht); 208 (Aruba)	139 RT-PCR positive (Utrecht); 63 RT-PCR positive (Aruba)	72.6 (Utrecht); 81.0 (Aruba)	100	[19]
Panbio COVID-19 Ag Rapid Test Device (Abbott Rapid Diagnostics, Cologne, Germany)	This assay is useful for mass testing where RT-PCR is not available particularly in asymptomatic.	341	182 symptomatic; 159asymptomatic	75.5	94.9	[20]
Panbio COVID-19 Ag Rapid Test Device (Abbott Rapid Diagnostics, Cologne, Germany)	This assay has good performance in suspected symptomatic in the first five days of symptom onset.	1369	140 RT-PCR positive	71.4	99.8	[21]
Panbio COVID-19 Ag Rapid Test Device (Abbott Rapid Diagnostics, Cologne, Germany)	Combination of saliva and NS could be used as alternative to NP when PPE is not available.	659	265 asymptomatic; 394 symptomatic	NA	NA	[22]
Rapid antigen test provided by R-Biopharm	This assay can detect individuals with high viral loads.	67 (Berlin); 70 (Frankfurt)	58 RT-PCR positive (Berlin); 32 RT-PCR positive (Berlin	77.6 (Berlin); 50 (Frankfurt)	100 (Berlin); 100 (Frankfurt)	[23]
STANDARD Q COVID-19 Ag (SD Biosensor, Korea)	This assay performed less than optimally.	262	90 RT-PCR positive	70	92	[24]
STANDARD Q COVID-19 Ag (SD Biosensor, Korea)	Adoption of POCTs in mass screening testing could decrease the burden on virology laboratories.	330	104 RT-PCR positive	70.6	100	[25]
STANDARD Q COVID-19 Ag (SD Biosensor, Korea)	This assay showed comparable sensitivity and specificity to that of RT-PCR.	454	60 RT-PCR positive	98.3	98.7	[26]
SD Biosensor SARS-CoV-2 Rapid Antigen Test (Roche Diagnostics, Basel, Switzerland)	This assay can detect mildly symptomatic cases during early phase.	970	NA	84.9	99.5	[27]
1. STANDARD Q COVID-19 Ag (SD Biosensor, Korea);<br>2. Espline SARS-CoV-2 (Fujirebio, Tokyo, Japan);<br>3. QuickNavi -COVID19 Ag (Denka Seiken, Tokyo, Japan);<br>4. ImmunoAce SARS-CoV-2 (Tauns Laboratories, Izunokuni, Japan)	The sensitivity of these four assays is lower than that of RT-qPCR but similar to that of virus isolation. Overall sensitivity of STANDARD Q COVID-19 Ag and Espline SARS-CoV-2 was better than that of ImmunoAce SARS-CoV-2 and QuickNavi COVID19 Ag.	76	76 RT-PCR positive (7 gargle lavages; 27 saliva; 2 throat swabs; 1 nasal vestibule swabs; 18 NS; 4 sputum; 17 tracheal aspirates)	NA	NA	[28]

STANDARD Q COVID-19 Ag (SD Biosensor, Korea)	Self-testing may enable wide-spread and more frequent testing.	303	39 RT-PCR positive	77.4 (self-testing); 79.5 (professional testing)	99.2 (self-testing); 99.6 (professional testing)	[29]
PANBIO COVID-19 Ag rapid test	The sensitivity of the assay increases with higher prevalence.	448	117 RT-PCR positive; 331 RT-PCR negative	85	100	[30]

*FDA-EUA; †These are preprint articles and have not been peer reviewed. NA, not available.

References

1. García-Fiñana M, Hughes DM, Cheyne CP, Burnside G, Stockbridge M, Fowler TA, et al. Performance of the Innova SARS-CoV-2 antigen rapid lateral flow test in the Liverpool asymptomatic testing pilot: population based cohort study. BMJ. 2021;374:n1637.

2. Van der Moeren N, Zwart VF, Lodder EB, Van den Bijllaardt W, Van Esch HRJM, Stohr JJJM, et al. Evaluation of the test accuracy of a SARS-CoV-2 rapid antigen test in symptomatic community dwelling individuals in the Netherlands. PLOS ONE. 2021;16(5):e0250886.

3. Perchetti GA, Huang M-L, Mills MG, Jerome KR, Greninger AL. Analytical Sensitivity of the Abbott BinaxNOW COVID-19 Ag CARD. Journal of Clinical Microbiology. 2020:JCM.02880-20.

4. Pilarowski G, Lebel P, Sunshine S, Liu J, Crawford E, Marquez C, et al. Performance characteristics of a rapid SARS-CoV-2 antigen detection assay at a public plaza testing site in San Francisco. medRxiv. 2020:2020.11.02.20223891.

5. Abdelrazik AM, Elshafie SM, Abdelaziz HM. Potential Use of Antigen-Based Rapid Test for SARS-CoV-2 in Respiratory Specimens in Low-Resource Settings in Egypt for Symptomatic Patients and High-Risk Contacts. Laboratory Medicine. 2020.

6. Corman VM, Haage VC, Bleicker T, Schmidt ML, Mühlemann B, Zuchowski M, et al. Comparison of seven commercial SARS-CoV-2 rapid point-of-care antigen tests: a single-centre laboratory evaluation study. The Lancet Microbe. 2021;2(7):e311-e9.

7. Weitzel T, Legarraga P, Iruretagoyena M, Pizarro G, Vollrath V, Araos R, et al. Comparative evaluation of four rapid SARS-CoV-2 antigen detection tests using universal transport medium. Travel Med Infect Dis. 2021;39:101942.

8. Porte L, Legarraga P, Vollrath V, Aguilera X, Munita JM, Araos R, et al. Evaluation of a novel antigen-based rapid detection test for the diagnosis of SARS-CoV-2 in respiratory samples. Int J Infect Dis. 2020;99:328-33.

9. Mertens P, De Vos N, Martiny D, Jassoy C, Mirazimi A, Cuypers L, et al. Development and Potential Usefulness of the COVID-19 Ag Respi-Strip Diagnostic Assay in a Pandemic Context. Front Med (Lausanne). 2020;7.

10. Lambert-Niclot S, Cuffel A, Le Pape S, Vauloup-Fellous C, Morand-Joubert L, Roque-Afonso A-M, et al. Evaluation of a Rapid Diagnostic Assay for Detection of SARS-CoV-2 Antigen in Nasopharyngeal Swabs. Journal of Clinical Microbiology. 2020;58(8):e00977-20.

11. Blairon L, Wilmet A, Beukinga I, Tré-Hardy M. Implementation of rapid SARS-CoV-2 antigenic testing in a laboratory without access to molecular methods: Experiences of a general hospital. J Clin Virol. 2020;129:104472-.

12. Scohy A, Anantharajah A, Bodéus M, Kabamba-Mukadi B, Verroken A, Rodriguez-Villalobos H. Low performance of rapid antigen detection test as frontline testing for COVID-19 diagnosis. J Clin Virol. 2020;129:104455-.

13. Mak GC, Lau SS, Wong KK, Chow NL, Lau CS, Lam ET, et al. Analytical sensitivity and clinical sensitivity of the three rapid antigen detection kits for detection of SARS-CoV-2 virus. J Clin Virol. 2020;133:104684-.

14. Courtellemont L, Guinard J, Guillaume C, Giaché S, Rzepecki V, Seve A, et al. High performance of a novel antigen detection test on nasopharyngeal specimens for diagnosing SARS-CoV-2 infection. Journal of Medical Virology. 2021;93(5):3152-7.

15. Ferguson J, Dunn S, Best A, Mirza J, Percival B, Mayhew M, et al. Validation testing to determine the sensitivity of lateral flow testing for asymptomatic SARS-CoV-2 detection in low prevalence settings: Testing frequency and public health messaging is key. PLOS Biology. 2021;19(4):e3001216.

16. Mak GCK, Lau SSY, Wong KKY, Chow NLS, Lau CS, Lam ETK, et al. Evaluation of rapid antigen detection kit from the WHO Emergency Use List for detecting SARS-CoV-2. J Clin Virol. 2020;134:104712-.

17. Albert E, Torres I, Bueno F, Huntley D, Molla E, Fernández-Fuentes MÁ, et al. Field evaluation of a rapid antigen test (Panbio™ COVID-19 Ag Rapid Test Device) for COVID-19 diagnosis in primary healthcare centres. Clinical Microbiology and Infection. 2020.

18. Linares M, Pérez-Tanoira R, Carrero A, Romanyk J, Pérez-García F, Gómez-Herruz P, et al. Panbio antigen rapid test is reliable to diagnose SARS-CoV-2 infection in the first 7 days after the onset of symptoms. J Clin Virol. 2020;133:104659-.

19. Gremmels H, Winkel BMF, Schuurman R, Rosingh A, Rigter NAM, Rodriguez O, et al. Real-life validation of the Panbio™ COVID-19 antigen rapid test (Abbott) in community-dwelling subjects with symptoms of potential SARS-CoV-2 infection. EClinicalMedicine. 2020.

20. Fenollar F, Bouam A, Ballouche M, Fuster L, Prudent E, Colson P, et al. Evaluation of the Panbio Covid-19 rapid antigen detection test device for the screening of patients with Covid-19. Journal of Clinical Microbiology. 2020:JCM.02589-20.

21. Bulilete O, Lorente P, Leiva A, Carandell E, Oliver A, Rojo E, et al. Panbio™ rapid antigen test for SARS-CoV-2 has acceptable accuracy in symptomatic patients in primary health care. Journal of Infection. 2021;82(3):391-8.

22. Agulló V, Fernández-González M, Ortiz de la Tabla V, Gonzalo-Jiménez N, García JA, Masiá M, et al. Evaluation of the rapid antigen test Panbio COVID-19 in saliva and nasal swabs in a population-based point-of-care study. J Infect. 2020:S0163-4453(20)30768-4.

23. Toptan T, Eckermann L, Pfeiffer AE, Hoehl S, Ciesek S, Drosten C, et al. Evaluation of a SARS-CoV-2 rapid antigen test: Potential to help reduce community spread? Journal of Clinical Virology. 2021;135:104713.

24. Nalumansi A, Lutalo T, Kayiwa J, Watera C, Balinandi S, Kiconco J, et al. Field evaluation of the performance of a SARS-CoV-2 antigen rapid diagnostic test in Uganda using nasopharyngeal samples. International Journal of Infectious Diseases. 2020.

25. Cerutti F, Burdino E, Milia MG, Allice T, Gregori G, Bruzzone B, et al. Urgent need of rapid tests for SARS CoV-2 antigen detection: Evaluation of the SD-Biosensor antigen test for SARS-CoV-2. J Clin Virol. 2020;132:104654-.

26. Chaimayo C, Kaewnaphan B, Tanlieng N, Athipanyasilp N, Sirijatuphat R, Chayakulkeeree M, et al. Rapid SARS-CoV-2 antigen detection assay in comparison with real-time RT-PCR assay for laboratory diagnosis of COVID-19 in Thailand. Virology Journal. 2020;17(1):177.

27. Igloi Z, Velzing J, van Beek J, van de Vijver D, Aron G, Ensing R, et al. Clinical Evaluation of Roche SD Biosensor Rapid Antigen Test for SARS-CoV-2 in Municipal Health Service Testing Site, the Netherlands. Emerging Infectious Disease journal. 2021;27(5):1323.

28. Yamayoshi S, Sakai-Tagawa Y, Koga M, Akasaka O, Nakachi I, Koh H, et al. Comparison of Rapid Antigen Tests for COVID-19. Viruses. 2020;12(12):1420.

29. Lindner AK, Nikolai O, Kausch F, Wintel M, Hommes F, Gertler MA-O, et al. Head-to-head comparison of SARS-CoV-2 antigen-detecting rapid test with self-collected nasal swab versus professional-collected nasopharyngeal swab. LID - 10.1183/13993003.03961-2020 [doi] LID - 2003961. Eur Respir J. 2021;57(4).

30. Escrivá BF, Mochón MDO, González RM, García CS, Pla AT, Ricart AS, et al. The effectiveness of rapid antigen test-based for SARS-CoV-2 detection in nursing homes in Valencia, Spain. Journal of Clinical Virology. 2021;143:104941.
