# Supplementary material for: Lateral Flow Immunoassays for SARS-CoV-2
Source: Diagnostics (Basel). 2022 Nov 18;12(11):2854. doi: 10.3390/diagnostics12112854 (PMC9689684; doi:10.3390/diagnostics12112854)
Supplement: Supplementary file 1 [file diagnostics-12-02854-s001.zip › Table S2.pdf]

Table S2. Comparison of performance evaluation between commercial serology LFIs for the detection of antibody against SARS-CoV-2.

**Test Name (Developer)**	**Main Findings**	***N***	**Subject Categorization**	**Immunogenic Protein**	**Sensitivity (%)**	**Specificity (%)**	**Reference**
BioMedomics COVID-19 IgG/IgM Rapid Antibody Test	The assay shows excellent sensitivity & specificity about 1-month post-symptom onset.	303	177 ELISA-positive; 43 ELISA-negative	RBD	90 (day 28)	100 (day 28)	[1]
1. Biosynex COVID-19 BSS (Biosynex, Switzerl&, Fribourg)<br>2. COVID-19 Sign IgM/IgG (Servibio/VEDALAB, France, Alençon)	Poor mutual agreement between LFI & ELISA.	325	198 RT-PCR positive; 127 controls	NA	1. 84<br>2. 65	89 (IgM); 100 (IgG)	[2]
1. NTBIO (NTBIO Diagnostics Inc., Surrey, British Columbia, Canada)<br>2. Orient-Gene (Zhejiang Orient-Gene Biotech Co. Ltd., Huzhou, China)<br>3. MEDsan (MEDsan GmbH, Biological Health Solutions, Hamburg, Germany)	These assays can be used when routine diagnostic serology facility is not available.	91	41 RT-PCR positive; 50 controls	NA	1. 92%<br>2. 87%<br>3. 100%	1. 100%<br>2. 98%<br>3. 98%	[3]
1. iCare Covid-19 Rapid Test (Covid-19 IgG/IgM Rapid test Kit) (Nantong Egens Biotechnology Co., Ltd, China);<br>2. Healgen COVID-19 IgG/IgM Rapid Test Cassette (Healgen Scientific Limited Liability Company, USA)*;<br>3. NADAL COVID-19 IgG/IgM Test (nal von minden GmbH, Germany);<br>4. BIOZEK Medical COVID-19 IgG/IgM Rapid Test Cassette (Inzec International Trading, The Netherl&s);<br>5. BIOSYNEX COVID −19 BSS (BIOSYNEX SWISS SA, Switzerl&);<br>6. Panbio COVID-19 IgG/IgM Rapid Test Device (Abbott Rapid Diagnostics Jena GmbH, Germany);<br>7. Acro 2019-nCoV IgG/IgM Rapid Teset(Acro Biotech Inc, USA);<br>8. ichroma COVID-19 Ab+ ichroma II instrument (Boditech Med Incorporated, Republic of Korea);	Only 7 assays are classified as "acceptable".	292	197 pre-pandemic; 65 convalescent plasma	1. S<br>2. S<br>3. N & S<br>4. N & S<br>5. S<br>6. N<br>7. N & S<br>8. N & S<br>9. N<br>10. N & S<br>11. NA<br>12. N<br>13. NA<br>14. S<br>15. N<br>16. NA<br>17. N & S<br>18. S<br>19. N & S<br>20. N & S<br>21. N<br>22. NA<br>23. N	IgM<br>1. 72.3<br>2. 67.7<br>3. 70.7<br>4. 15.4<br>5. 73.8<br>6. 9.2<br>7. 15.4<br>8. 4.6<br>9. 20<br>10. 55.4<br>11. 46.2<br>12. 63.1<br>13. 67.7<br>15. 81.5<br>16. 69.2<br>17. 78.5<br>18. 16.9<br>19. 96.9<br>20. 76.9<br>22. 60<br>23. 47.6<br>24. 55.4	IgM<br>1. 89.3<br>2.99<br>3. 98<br>4. 96.4<br>5. 95.9<br>6. 98<br>7. 95.4<br>8. 99.5<br>9. 97<br>10. 99.5<br>11. 95.9<br>12. 96.4<br>13. 95.4<br>15. 94.9<br>16. 98<br>17. 80.8<br>18. 99.5<br>19. 82.1<br>20. 92.8<br>22. 97.9<br>23. 95.2<br>24. 96.4	[4]

9. COVID-19 IgG-IgM Rapid test	24. S	25. 20	25. 96.9
(DIASource ImmunoAssays S.A.,	25. N	26. 24.6	26. 98.5
Belgium);	26. N	27. 27.7	27. 98.5
10. Diagnostic Kit for IgM/IgG Antibody	27. N, S & E	28. 75.4	28. 96.9
to Coronavirus (SARS-CoV-2) (Lateral	28. N & S	29. 73.8	29. 94.3
Flow) (Zhuhai Livzon Diagnostics Inc.,	29. NA	31. 56.9	31. 98.9
China);	30. N		
11. COVISURE™ COVID-19 IgG-IgM	31. N	IgG	IgG
Rapid Test (W.H.P.M. Biosearch &	32. S	1. 84.6	1. 90.9
Technology Co.,Ltd., China);		2. 98.5	2. 99
12. ST&ARD Q COVID-19 IgM/IgG		3. 90.8	3. 99.5
Combo Test (SD Biosensor, Republic of		4. 92.3	4. 99
Korea);		5. 84.6	5. 100
13. Novel Coronavirus (2019-nCoV)		6. 78.5	6. 100
IgG/IgM Test Kit (Colloidal gold)		7. 87.7	7. 99
(Genrui Biotech Inc., China);		8. 92.3	8. 95.9
14. WANTAI SARS-CoV-2 Ab Rapid		9. 81.5	9. 98.5
Test (Beijing Wantai Biological		10. 60	10. 100
Pharmacy Enterprise Co., Ltd.,		11. 58.5	11. 95.9
China)*;		12. 98.5	12. 98.5
15. Leccurate SARS-CoV-2 Antibody Test		13. 75.4	13. 99.5
Kit (Beijing Lepu Medical Technology		15. 87.7	15. 98.5
Co., Ltd., China);		16. 92.3	16. 98
16. OnSite Covid-19 IgG/IgM (CTK		17. 96.9	17. 91.9
Biotech, USA);		18. 90.8	18. 92.9
17. COVID-19 IgG/IgM Rapid Test Kit		19. 78.5	19. 99.5
(Abbexa Ltd, UK);		20. 66.2	20. 99
18. Anti-SARS-CoV-2 Rapid Test		22. 53.8	22. 98
(AutoBio Diagnostics);		23. 92.1	23. 95.2
19. Instant-View COVID-19 IgG/IgM		24. 96.9	24. 96.9
Antibody Test (Alfa Scientific Designs,		25. 84.6	25. 98.5
Inc. USA);		26. 78.5	26. 98.5
20. 2019-nCOV IgG/IgM rapid test		27. 86.2	27. 97
(Dynamiker Biotechnology (Tianjin)		28. 67.7	28. 99.5
Co., Ltd., China);		29. 83.1	29. 98.5
21. INgezim COVID 19 CROM		31. 95.4	31. 98.9
(kassett)(Inmunología y Genética			
Aplicada, S.A. (INGENASA), Spain);		14. 83.1	14. 98
22. SARS -CoV-2 IgM/IgG Antibody		21. 80	21. 99.5
Detection Kit (HONGKONG SENTE		30. 64.6	30. 91.7
		32. 100	32. 99.5

INDUSTRIAL INTERNATIONAL TRADE CO., LIMITED, China);<br>23. COVID19 IgG & IgM Test Kit (colloidal gold method) (Zhejiang Anji Saianfu Biotech Co.,Ltd., China);<br>24. COVID-19 IgG/IgM Rapid Test (Hangzhou AllTest Biotech Co., Ltd., China);<br>25. 2019-nCovid IgG/IgM Rapid Test Cassette (BioMaxima);<br>26. Diagnostic Kit for SARS-Cov-2 IgM/IgG Antibody (Collodial Gold) (Shanghai Kehua Biological Engineering Co., Ltd.);<br>27. nCOVID-19 IgG & IgM POCT (Technogenetics S.r.l, Italy);<br>28. StrongStep® COVID-19 IgG/IgM Combo Test (NanJing Liming Bio-products Co. Ltd.);<br>29. COVID-19 IgG/IgM RAPID TEST (ASSUT EUROPE SPA);<br>30. EBS Alert SARS-CoV-2 ANTIBODY RAPID TEST (Excelsior Bio-System Incorporation);<br>31. Chembio DPP COVID-19 IgM/IgG System 2.0 (Chembio Diag. Systems Inc);<br>32. LumiraDx SARS-CoV-2 Ab Test (LumiraDx UK Ltd)							
1. API<br>2. API (v2)<br>3. BioHit*<br>4. BTNX<br>5. Camtech<br>6. CareHealth<br>7. Cellex<br>8. Edinburgh<br>9. Genobio<br>10. InTec<br>11. KHB<br>12. Lumiquick	The assays' performance varied with tests.	112	56 pre-pandemic; 56 SARS-CoV-2 positive	1. N & S<br>2. N & S<br>3. N & S<br>4. N & S<br>5. S<br>6. N<br>7. N & S<br>8. N & S<br>9. N & S<br>10. N & S<br>11. N<br>12. N & S	1. 64.3<br>2. 91.1<br>3. 94.6<br>4. 87<br>5. 78.6<br>6. 91.1<br>7. 82.1<br>8. 63.6<br>9. 70.9<br>10. 82.1<br>11. 69.6<br>12. 72.7	1. 100<br>2. 100<br>3. 92.6<br>4. 100<br>5. 100<br>6. 100<br>7. 98.2<br>8. 96.2<br>9. 100<br>10. 98.2<br>11. 100<br>12. 100	[5]

13. Oranoxis<br>14. OZO<br>15. Phamatech<br>16. Ray Biotech<br>17. Ray Biotech (v2)<br>18. U2U<br>19. Vivachek<br>20. Zhuhai Livzon				13. S<br>14. S<br>15. N<br>16. N<br>17. N<br>18. N & S<br>19. N & S<br>20. N & S	13. 78.2<br>14. 33.9<br>15. 75<br>16. 73.2<br>17. 85.7<br>18. 39.3<br>19. 94.5<br>20. 81.8	13. 100<br>14. 100<br>15. 100<br>16. 100<br>17. 100<br>18. 100<br>19. 98.2<br>20. 100	
1. Biosensor<br>2. AMS International<br>3. Leccurate<br>4. HIGHTOP One Step rapid test<br>5. Cromatest COVID-19<br>6. AMP Rapid Test<br>7. Egens<br>8. Cellex<br>9. Onesite Rapidtest	These assays should not be used as a diagnostic test but can be used to complement RT-PCR.	293	36 asymptomatic; 57 symptomatic; 100 negatives	NA	IgM<br>1. 91.7<br>2. 95.7<br>3. 90<br>4. 82<br>5. 78.03<br>6. 95.7<br>7. 96.88<br>8. 93.75<br>9. 78.03<br><br>IgG<br>1. 79.2<br>2. 91.8<br>3. NR<br>4. 93<br>5. 96.86<br>6. 91.8<br>7. 96.88<br>8. 93.75<br>9. 96.86	IgM<br>1. 100<br>2. 97.3<br>3. 100<br>4. 96<br>5. 99.39<br>6. 97.3<br>7. 100<br>8. 96.4<br>9. 99.39<br><br>IgG<br>1. 100<br>2. 96.4<br>3. NR<br>4. 97.5<br>5. 100<br>6. 96.4<br>7. 100<br>8. 96.4<br>9. 100	[6]
1. OnSite COVID-19 IgG/IgM Rapid Test (CTK Biotech Inc., CA, USA)<br>2. VivaDiag COVID-19 IgM/IgG Rapid Test (VivaChek, Wilmington, USA)<br>3. Hangzhou AllTest COVID-19 test (Hangzhou AllTest Biotech, Hangzhou, China)<br>4. Wondfo SARS-CoV-2 Antibody Test (Guangzhou Wondfo Biotech, Guagzhou, China)<br>5. Hightop SARS-CoV-2 IgM/IgG Antibody Rapid Test	These assays should not be used as a diagnostic test.	183	91 RT-PCR positive	NA	1. 1. 84.6<br>2. 2. 78.5<br>3. 3. 90.8<br>4. 4. 93.8<br>5. 5. 93.8	1. 95.6<br>2. 97.8<br>3. 96.7<br>4. 97<br>5. 100	[7]

1. 2019-nCoV IgG/IgM Rapid Test (Acro Biotech Inc., CA, USA)<br>2. Anti-SARS-CoV-2 Rapid Test (Autobio Diagnostics Co. Ltd, Zhengzhou, China)<br>3. COVID-19 IgG/IgM Rapid Test (Healgen Scientific Limited Liability Company, Houston, TX, USA/Zhejiang Orient Gene Biotech Co. Ltd, Zhejiang, China)*<br>4. NADAL COVID-19 IgG/IgM Test (Nal von Minden GmbH, Moers, Germany)<br>5. OnSite COVID-19 IgG/IgM Rapid Test (CTK Biotech Inc., CA, USA)	These assays have difficulty in detecting samples with low IgG titer which must be taken into considerations when choosing antibody tests.	183	87 RT-PCR positive; 96 RT-PCR negative	1. NA<br>2. S<br>3. S1<br>4. NA<br>5. S1	1. 78.2<br>2. 58.6<br>3. 81.6<br>4. 56.3<br>5. 67.8	99 (for all)	[8]
1. Accu-Tell COVID-19 IgG/IgM Rapid Test (AccuBioTech, Beijing, China);<br>2. The Diagnostic Kit for IgM / IgG Antibody to Coronavirus (SARS-CoV-2) (Lateral Flow) (Zhuhai Livzon Diagnostics, Zhuhai, China);<br>3. Coronavirus (COVID-19) IgM/IgG Rapid Test Kit (ISIA BIO-Technology, Chongqing, China);<br>4. H2019-nCoV IgM Antibody Test Kit (Hecin Biotech Co., Ltd., Guangzhou, China)<br>5. COVID-19 IgG/IgM Rapid Test Kit (UNscience Biotechnology, Wuhan, China);<br>6. 2019-nCoV IgM/IgG Rapid Test (Acro Biotech, CA, USA)	These assays have a good capacity to detect SARS-CoV-2 antibodies two weeks post-symptom onset.	58	38 RT-PCR positive; 20 controls	NA	IgM<br>1. 91.7<br>2. 80<br>3. 93.3<br>4. 86.7<br>5. 80<br>6. 76.9<br><br>IgG<br>1. 84.6<br>2. 86.7<br>3. 80<br>4. 86.7<br>5. 84.6	IgM<br>1. 100<br>2. 95<br>3. 100<br>4. 100<br>5. 65<br>6. 100<br><br>IgG<br>1. 100<br>2. 100<br>3. 100<br>4. 95<br>5. 100	[9]
AllTest 2019-nCoV IgG/IgM Rapid Test Cassette (Hangzhou AllTest Biotech, Hangzhou, China)	Antibody response varied with different clinical manifestations & disease severity. Patients with symptoms & IgM positive have a shorter duration of viral shedding & no worsening clinical conditions.	61	14 RT-PCR positive; 28 controls	N	78.6	100	[10]

AllTest 2019-nCoV IgG/IgM Rapid Test Cassette (Hangzhou AllTest Biotech, Hangzhou, China)	This assay was a reliable tool for diagnosis SARS-CoV-2 infection from 14 days post-symptomatic especially among RT-PCR negative patients.	251	90 RT-PCR positive; 161 RT-PCR negative	N	64.4	100	[11]
Colloidal gold immunochromatography antibody detection kit (Shanghai Outdo Biotech, China)	This assay has the potential for use as a rapid diagnosis test for COVID-19	150	150 suspected cases; 97 RT-PCR positive	S, M & N	71.1	96.2	[12]
COVID-19 IgG/IgM Rapid Test Cassette (Zhejiang Orient Gene Biotech, Huzhou, China)	This assay is suitable for assessing previous exposure. Negative results may be unreliable during the first weeks after infection.	153	29 RT-PCR positive; 124 controls	NA	69.0 (IgM)<br>93.1 (IgG)	100 (IgM)<br>99.2 (IgG)	[13]
COVID-19 IgG/IgM Rapid Test Cassette (Zhejiang Orient Gene Biotech, Huzhou, China)	This assay is suitable for routine use for patients with onset of symptoms ≥ 10 days ago.	148	102 RT-PCR positive	NA	95.8	100	[14]
Livzon IgM/IgG Diagnostic Kit for IgM/IgG Antibody to Coronovirus (SARS-Cov-2) Lateral Flow (Zhuhai Livzon Diagnostics, Zhuhai, China)	IgM-positive rate increased from 11.1% in early-stage to 74.2% in late-stage disease. IgG-positive rate increased from 3.6% in early-stage to 96.8% in late-stage disease.	86	67 RT-PCR positive	NA	11.1 (< 7days)<br>92.9 (7-14 days)<br>96.8 (14 days)	NA	[15]
One Step Novel Coronavirus (COVID-19) IgM/IgG Antibody Test (Artron, Burnaby, Canada)	This assay is not recommended as initial COVID-19 diagnostic test.	186	112 RT-PCR positive	NA	43.2	98	[16]
1. Rapid SARS -CoV-2 Antibody (IgM/IgG) Test (InTec, Xiamen, China);<br>2. qSARS-CoV-2 IgG/IgM Cassette Rapid Test (GICA) (Cellex, NC, USA)*;<br>3. COVID-19 IgG/IgM Rapid Test Cassette (Healgen Scientific, Houston, USA)*	These assays have sufficient PPV in high seroprevalence scenarios (≥ 50%). They might have a role for diagnostic among individual with a sufficiently high pretest probability to supplement RT-PCR. They are suitable for use in general population to rule out the presence of SARS-CoV-2 antibodies.	334	107 RT-PCR positive	1. N<br>2. S & N<br>3. S & N	1. 90<br>2. 80<br>3. 100	1. 85<br>2. 99<br>3. 88	[17]
SARS-CoV-2 IgG/IgM antibody test kit (Innovita Biological Technology Co., Ltd, Tangshan, Hebei, China)	This assay can be used to complement RT-PCR.	179	90 RT-PCR positive; 89 RT-PCR negative	NA	85.6	91	[18]

The Diagnostic Kit for IgM / IgG Antibody to Coronavirus (SARS-CoV-2) (Lateral Flow) (Zhuhai Livzon Diagnostics, Zhuhai, China)	This assay can be used for clinical reference.	189	154 patients; 35 controls	NA	82.4	100	[19] †
VivaDiag COVID-19 IgM/IgG Rapid Test (VivaChek, Wilmington, USA)	Not recommended for triage of patients with suspected COVID-19.	110	30 RT-PCR positive; 50 with respiratory symptoms; 30 RT-PCR negative	RBD	18.4	91.7	[20]
VivaDiag COVID-19 IgM/IgG Rapid Test (VivaChek, Wilmington, USA)	This assay provides information on immunoreaction of individuals.	191	191 symptomatic patients	RBD	30	89	[21]
2019-nCoV IgG/IgM Antibody Rapid Test Kit (Beijing Diagreat Biotechnologies, Beijing, China)	This assay is quite reliable & useful.	41	27 RT-PCR positive; 7 symptomatic RT-PCR negative; 7 controls	NA	83	93	[22]
2019-nCoV Ab Test (Colloidal Gold) (Innovita Biological Technology, Beijing, China)*	This assay can be used as an effective supplementary indicator for suspected cases with negative RT-PCR results.	76	38 symptomatic patients	NA	50 (IgM)<br>92.1 (IgG)	NA	[23]
1. 2019-nCoV Ab Test (Colloidal Gold) (Innovita Biological Technology, Beijing, China)*;<br>2. SD Biosensor Test (SD Biosensor, Korea);<br>3. Wondfo test (Guangzhou Wondfo Biotech, Guagzhou, China)<br>4. Runkun test (Runkun Pharmaceutical, Hunan, China)		1195	570 symptomatic; 625 asymptomatic	NA	1. 93<br>2. 94<br>3. 98<br>4. 76	1. 49<br>2. 69<br>3. 75<br>4. 69	[24]
1. 2019-n-CoV IgG/IgM rapid test cassette (Bio Marketing Diagnostics, Akiva, Israel);<br>2. Novel Coronavirus (2019-n-CoV) antibody IgG/IgM assay (colloidal gold) (Avioq, Bio-Tech, Sh&ong, China);<br>3. QuickZen COVID-19 IgM/IgG Kit (ZenTech, Angleur, Belgium)	Overall sensitivity was similar (~70 %). Sensitivity increased during the second week after post-symptomatic onset (91-94%).	400	128 RT-PCR positive; 272 controls	NA	1. 71.9<br>2. 68.8<br>3. 71.1	1. 100<br>2. 95.8<br>3. 100	[25]

NA, not available.

References

1. Churiwal M, Lin KD, Khan S, Chhetri S, Muller MS, Tompkins K, et al. Assessment of the Field Utility of a Rapid Point-of-Care Test for SARS-CoV-2 Antibodies in a Household Cohort. The American Journal of Tropical Medicine and Hygiene. 2022;106(1):156-9.
2. Velay A, Gallais F, Benotmane I, Wendling MJ, Danion F, Collange O, et al. Evaluation of the performance of SARS-CoV-2 serological tools and their positioning in COVID-19 diagnostic strategies. Diagnostic Microbiology and Infectious Disease. 2020;98(4):115181.
3. Andrey DO, Cohen P, Meyer B, Torriani G, Yerly S, Mazza L, et al. Head-to-Head Accuracy Comparison of Three Commercial COVID-19 IgM/IgG Serology Rapid Tests. Journal of Clinical Medicine [Internet]. 2020; 9(8).
4. Tollånes MC, Jenum PA, Kierkegaard H, Abildsnes E, Bævre-Jensen RM, Breivik AC, et al. Evaluation of 32 rapid tests for detection of antibodies against SARS-CoV-2. Clinica Chimica Acta. 2021;519:133-9.
5. Trombetta BA, Kandigian SE, Kitchen RR, Grauwet K, Webb PK, Miller GA, et al. Evaluation of serological lateral flow assays for severe acute respiratory syndrome coronavirus-2. BMC Infectious Diseases. 2021;21(1):580.
6. Mercado M, Malagón-Rojas J, Delgado G, Rubio VV, Muñoz Galindo L, Parra Barrera EL, et al. Evaluation of nine serological rapid tests for the detection of SARS-CoV-2. Rev Panam Salud Publica. 2020;44:e149.
7. Bond K, Nicholson S, Lim SM, Karapanagiotidis T, Williams E, Johnson D, et al. Evaluation of Serological Tests for SARS-CoV-2: Implications for Serology Testing in a Low-Prevalence Setting. The Journal of Infectious Diseases. 2020;222(8):1280-8.
8. Lagerqvist N, Maleki KT, Verner-Carlsson J, Olausson M, Dillner J, Wigren Byström J, et al. Evaluation of 11 SARS-CoV-2 antibody tests by using samples from patients with defined IgG antibody titers. Scientific Reports. 2021;11(1):7614.
9. Tuaillon E, Bolloré K, Pisoni A, Debiesse S, Renault C, Marie S, et al. Detection of SARS-CoV-2 antibodies using commercial assays and seroconversion patterns in hospitalized patients. Journal of Infection. 2020;81(2):e39-e45.
10. Lee Y-L, Liao C-H, Liu P-Y, Cheng C-Y, Chung M-Y, Liu C-E, et al. Dynamics of anti-SARS-Cov-2 IgM and IgG antibodies among COVID-19 patients. J Infect. 2020;81(2):e55-e8.
11. Pérez-García F, Pérez-Tanoira R, Romanyk J, Arroyo T, Gómez-Herruz P, Cuadros-González J. Alltest rapid lateral flow immunoassays is reliable in diagnosing SARS-CoV-2 infection from 14 days after symptom onset: A prospective single-center study. Journal of Clinical Virology. 2020;129:104473.
12. Shen B, Zheng Y, Zhang X, Zhang W, Wang D, Jin J, et al. Clinical evaluation of a rapid colloidal gold immunochromatography assay for SARS-Cov-2 IgM/IgG. Am J Transl Res. 2020;12(4):1348-54.
13. Hoffman T, Nissen K, Krambrich J, Rönnberg B, Akaberi D, Esmaeilzadeh M, et al. Evaluation of a COVID-19 IgM and IgG rapid test; an efficient tool for assessment of past exposure to SARS-CoV-2. Infection Ecology & Epidemiology. 2020;10(1):1754538.
14. Dellière S, Salmona M, Minier M, Gabassi A, Alanio A, Le Goff J, et al. Evaluation of the COVID-19 IgG/IgM Rapid Test from Orient Gene Biotech. Journal of Clinical Microbiology. 2020;58(8):e01233-20.

15. Pan Y, Li X, Yang G, Fan J, Tang Y, Zhao J, et al. Serological immunochromatographic approach in diagnosis with SARS-CoV-2 infected COVID-19 patients. Journal of Infection. 2020;81(1):e28-e32.

16. Imai K, Tabata S, Ikeda M, Noguchi S, Kitagawa Y, Matuoka M, et al. Clinical evaluation of an immunochromatographic IgM/IgG antibody assay and chest computed tomography for the diagnosis of COVID-19. Journal of Clinical Virology. 2020;128:104393.

17. GeurtsvanKessel CH, Okba NMA, Igloi Z, Bogers S, Embregts CWE, Laksono BM, et al. An evaluation of COVID-19 serological assays informs future diagnostics and exposure assessment. Nature Communications. 2020;11(1):3436.

18. Liu Y, Liu Y-P, Diao B, Ding J-Y, Yuan M-X, Ren F-F, et al. Diagnostic indexes of a rapid immunoglobulin G/immunoglobulin M combined antibody test for severe acute respiratory syndrome coronavirus 2. Chinese Medical Journal. 2021;134(4).

19. Xiang J, Yan M, Li H, Liu T, Lin C, Huang S, et al. Evaluation of Enzyme-Linked Immunoassay and Colloidal Gold-Immunochromatographic Assay Kit for Detection of Novel Coronavirus (SARS-Cov-2) Causing an Outbreak of Pneumonia (COVID-19). medRxiv. 2020:2020.02.27.20028787.

20. Cassaniti I, Novazzi F, Giardina F, Salinaro F, Sachs M, Perlini S, et al. Performance of VivaDiag COVID-19 IgM/IgG Rapid Test is inadequate for diagnosis of COVID-19 in acute patients referring to emergency room department. Journal of Medical Virology. 2020;92(10):1724-7.

21. Paradiso AV, De Summa S, Loconsole D, Procacci V, Sallustio A, Centrone F, et al. Rapid Serological Assays and SARS-CoV-2 Real-Time Polymerase Chain Reaction Assays for the Detection of SARS-CoV-2: Comparative Study. J Med Internet Res. 2020;22(10):e19152.

22. Spicuzza L, Montineri A, Manuele R, Crimi C, Pistorio MP, Campisi R, et al. Reliability and usefulness of a rapid IgM‐IgG antibody test for the diagnosis of SARS-CoV-2 infection: A preliminary report. Journal of Infection. 2020;81(2):e53-e4.

23. Yong G, Yi Y, Tuantuan L, Xiaowu W, Xiuyong L, Ang L, et al. Evaluation of the auxiliary diagnostic value of antibody assays for the detection of novel coronavirus (SARS-CoV-2). Journal of Medical Virology. 2020;92(10):1975-9.

24. Boum Y, Fai KN, Nicolay B, Mboringong AB, Bebell LM, Ndifon M, et al. Performance and operational feasibility of antigen and antibody rapid diagnostic tests for COVID-19 in symptomatic and asymptomatic patients in Cameroon: a clinical, prospective, diagnostic accuracy study. The Lancet Infectious Diseases. 2021.

25. Montesinos I, Gruson D, Kabamba B, Dahma H, Van den Wijngaert S, Reza S, et al. Evaluation of two automated and three rapid lateral flow immunoassays for the detection of anti-SARS-CoV-2 antibodies. Journal of Clinical Virology. 2020;128:104413.
